# Host Tissue and Glycan Binding Specificities of Avian Viral Attachment Proteins Using Novel Avian Tissue Microarrays

**DOI:** 10.1371/journal.pone.0128893

**Published:** 2015-06-02

**Authors:** Iresha N. Ambepitiya Wickramasinghe, Robert P. de Vries, Amber M. Eggert, Nantaporn Wandee, Cornelis A. M. de Haan, Andrea Gröne, Monique H. Verheije

**Affiliations:** 1 Department of Pathobiology, Faculty of Veterinary Medicine, Utrecht University, Utrecht, The Netherlands; 2 Department of Infectious Diseases & Immunology, Faculty of Veterinary Medicine, Utrecht University, Utrecht, The Netherlands; Deutsches Primatenzentrum GmbH—Leibniz-Institut fur Primatenforschung, GERMANY

## Abstract

The initial interaction between viral attachment proteins and the host cell is a critical determinant for the susceptibility of a host for a particular virus. To increase our understanding of avian pathogens and the susceptibility of poultry species, we developed novel avian tissue microarrays (TMAs). Tissue binding profiles of avian viral attachment proteins were studied by performing histochemistry on multi-species TMA, comprising of selected tissues from ten avian species, and single-species TMAs, grouping organ systems of each species together. The attachment pattern of the hemagglutinin protein was in line with the reported tropism of influenza virus H5N1, confirming the validity of TMAs in profiling the initial virus-host interaction. The previously believed chicken-specific coronavirus (CoV) M41 spike (S1) protein displayed a broad attachment pattern to respiratory tissues of various avian species, albeit with lower affinity than hemagglutinin, suggesting that other avian species might be susceptible for chicken CoV. When comparing tissue-specific binding patterns of various avian coronaviral S1 proteins on the single-species TMAs, chicken and partridge CoV S1 had predominant affinity for the trachea, while pigeon CoV S1 showed marked preference for lung of their respective hosts. Binding of all coronaviral S1 proteins was dependent on sialic acids; however, while chicken CoV S1 preferred sialic acids type I lactosamine (Gal(1-3)GlcNAc) over type II (Gal(1-4)GlcNAc), the fine glycan specificities of pigeon and partridge CoVs were different, as chicken CoV S1-specific sialylglycopolymers could not block their binding to tissues. Taken together, TMAs provide a novel platform in the field of infectious diseases to allow identification of binding specificities of viral attachment proteins and are helpful to gain insight into the susceptibility of host and organ for avian pathogens.

## Introduction

Viral infection of birds can vary from asymptomatic to severe clinical disease. In commercial birds viral diseases can have a large impact on the welfare of the animal as well as the production of eggs and meat. In addition, clinically asymptomatic infected birds may be a threat to the environment by becoming a reservoir for various avian viruses, including influenza A virus (IAV) [1]. For many avian viruses, in particular for the avian gammacoronaviruses (CoV), hardly anything is known about the specific interactions between virus and host determining the outcome of the infection. Elucidation of such viral or host determinants for predilection of organ system, or particular avian species, to these viruses is hampered by the lack of infection model systems. Novel assays are therefore needed to ultimately to better control of virus infections in susceptible birds.

Avian gammacoronaviruses, belonging to the family of *Coronaviridae* within the order *Nidovirales*, are represented by infectious bronchitis virus (IBV), the coronavirus of chicken *(Gallus gallus)*. Infectious bronchitis is a highly contagious disease, causing huge economic losses worldwide. While all chicken CoV strains infect epithelial cells of the respiratory system [2] some IBVs have a preference for other organ systems including the urogenital system [3]. Limited knowledge is available on which specific viral and host factors determine the susceptibility of specific epithelial cells within the chicken organ systems. In addition, it is unknown whether IBV strains, usually considered as chicken-specific, can cross the species barrier to infect, and cause disease, in other birds. Gammacoronaviruses have also been detected in other poultry species, including turkey, pheasant, pigeon, partridge, guineafowl, quail, goose, teal, peafowl [2], [4–7]. Some of these have high sequence similarity to IBV (>90%, [7]) and are sometimes referred to IBV-like strains [2].

The coronaviral glycoprotein spike (S), residing in the viral envelope, is the major viral attachment protein and the determinant for the *in vitro* cell tropism of IBV [8]. The high sequence diversity between various IBV (-like) strains [2] suggests that it might contribute to the outcome of the infection *in vivo* [9], but likely it is not the only determinant for the pathogenicity [10]. The spike protein binds to sialic acids, in particular, α2,3-linked sialylated glycans [11] on the cell surface of susceptible host cells [11–13] and thus might contribute to the tissue and host tropism of the virus. However, as sialic acids are distributed universally in host tissues [14–16], combined with the observation that chicken CoV infects only particular cells and organ systems [3], it has been suggested that also other host factors contribute to the tropism. This might be an additional specific protein receptor, linkage of sialic acids to a particular protein or lipid, or another essential entry factor.

Distribution of viral receptors in avian species has been studied extensively to compare the binding with the *in vivo* tropism and pathogenicity of that particular virus. For example, while sialic acid distribution in the host tissues has been shown to correlate with tissue attachment patterns of labeled virus [15], binding of viral attachment proteins to tissues reflected the *in vivo* pathogenicity of influenza virus [17]. Such a tissue-based approach therefore, can be taken into account to elucidate multiple host attachment factors and thus to facilitate the prediction of the susceptibility of an organ system or a host species to a virus. Previously others and we have shown that binding of recombinant coronaviral attachment proteins to host tissues can be used to elucidate tissue and glycan binding specificities of avian coronaviruses [11], [18], [19]. In particular, binding of the spike proteins from IBV strains could be correlated with reported pathogenicity of the virus [11].

To expand our knowledge on the host and organ tropism of avian gammacoronaviruses, we aimed at profiling tissue attachment characteristics of various IBV(-like) spike proteins. However, analyzing the binding of viral components on tissues, from a range of host or organ systems, mounted on multiple microscopic slides is laborious and demands ample amounts of labeled viral components. To facilitate this analysis we developed tissue microarrays (TMAs), representing tissues from ten different avian species, from which gammacoronaviral sequences were detected [2]. Multi-species and single-species TMAs were developed, by transferring 2 mm tissue cores from archive blocks to recipient array blocks. First, tissue attachment characteristics of the attachment protein hemagglutinin (HA) of influenza virus H5N1 and the spike protein of avian CoVs were compared on respiratory tissues of different avian species using multi-species TMA. Next, single-species TMA was used to study tissue and glycan binding profiles of spike proteins of various avian coronaviruses, including those detected in chicken, pigeon and partridge CoVs. We observed that recombinant spike protein of the prototype chicken CoV strain M41 could attach to respiratory tissues of various other avian species albeit with lower avidity than that observed for HA. Spike proteins of pigeon CoV and partridge CoV also displayed sialic acid specific tissue binding, however, their fine receptor specificity was not identical to that of the IBV M41 spike. In conclusion, TMAs are excellent tools to gain insight in virus-host interactions involved in the first step of the infection.

## Materials and Methods

### Ethics statement

The tissues used for this study were obtained from the tissue archive of the Veterinary Pathologic Diagnostic Center (Department of Pathobiology, Faculty of Veterinary Medicine, Utrecht University, The Netherlands). This archive is composed of paraffin blocks with tissues maintained for diagnostic purposes; no permission of the Committee on the Ethics of Animal Experiment is required.

### Animals and tissues

Tissues were selected from Canada goose, graylag goose, guineafowl, mallard duck, partridge, pheasant, pigeon, quail, teal, turkey, and white leghorn chicken. The donor blocks were sectioned to 3–4 μm. Sections were stained with hematoxylin and eosin (H&E) assessed microscopically to analyze the quality of preservation and to select areas from organ systems devoid of pathological changes for transfer into recipient blocks.

### Preparation of tissue microarray (TMA)

**Fig 1:**
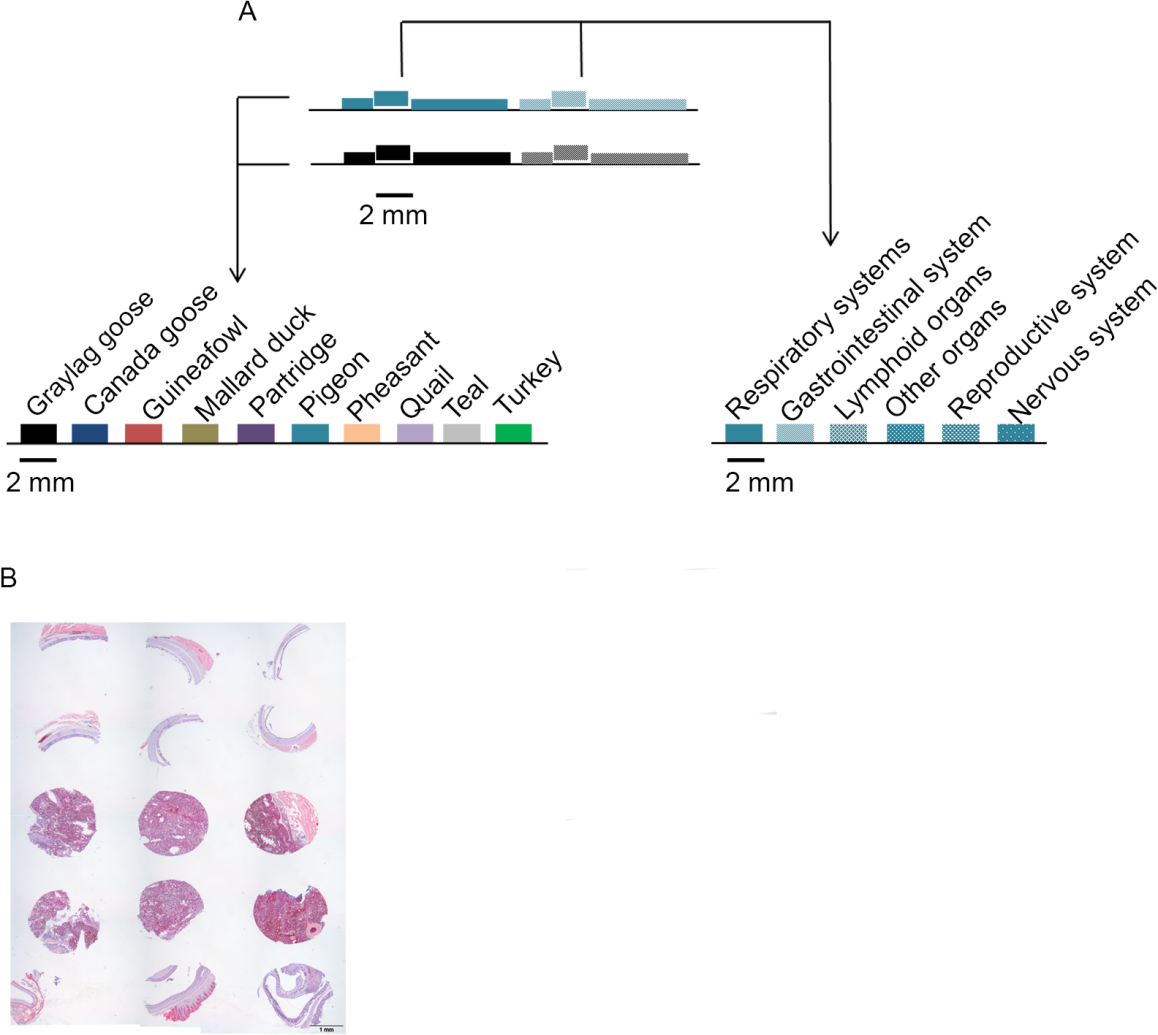
Development of avian tissue microarrays (TMA). (A) Tissue cores from similar organs from ten avian species were grouped in multispecies TMAs and tissues from one species were grouped in single-species TMA; (B) representative example of arrangement of tissues in multi-species TMA stained with H&E.

The recipient array block, containing 60 holes of 2 mm diameter, was prepared by first pouring paraplast X-TRA (Sigma, Aldrich, The Netherlands) into an array mold (IHC world, USA). Selected cores from the donor blocks were subsequently transferred to the recipient array block (Fig 1). The array blocks were trimmed and sectioned into 4 μm TMA sections mounted on KP plus glass slides (Klinipath, The Netherlands). A TMA from every array block was stained with H&E and lectins MALI (*Maackia amurensis* lectin I) and MALII (*Maackia amurensis* lectin II) (Vector Laboratories) for quality control and to identify the different cell types in each tissue.

### Genes and expression vectors

S1 encoding sequences of pigeon and partridge CoVs [GenBank: AAZ85066 and AAT70772 respectively] were obtained from the National Center for Biotechnology Information (NCBI). The pCD5 expression vectors containing S1 of chicken coronavirus, strain M41 S1 and (HA) of avian influenza virus are previously described [11]. To generate pCD5 expression vectors containing S1 of pigeon and partridge CoVs codon optimized S1 sequences were obtained from GenScript (USA) and cloned using the introduced upstream *Nhe*I and downstream *Pac*I restriction sites into pCD5 expression plasmid by restriction digestion. The N- terminal CD5 signal peptide was followed by the S1 gene, and c- terminal GCN4 trimerization domain (GCN4; RMKQIEDKIEEIESKQKKIENEIARIKKLVPRGSLE) and the *Strep*-Tag II (ST;WSHPQFEK, IBA GmbH).

### Expression and purification of proteins

Recombinant S1 proteins were expressed and purified as previously described [11]. In short, pCD5 vectors containing S1/HA domains were transfected into human embryo kidney cells and cell culture supernatants were harvested after 7 days. S1 proteins were purified by adding *Strep*-Tactin sepharose 50% suspension (IBA GmbH) and analyzed in SDS-PAGE followed by western blotting and gel code blue staining.

### Protein histochemistry

**Fig 2:**
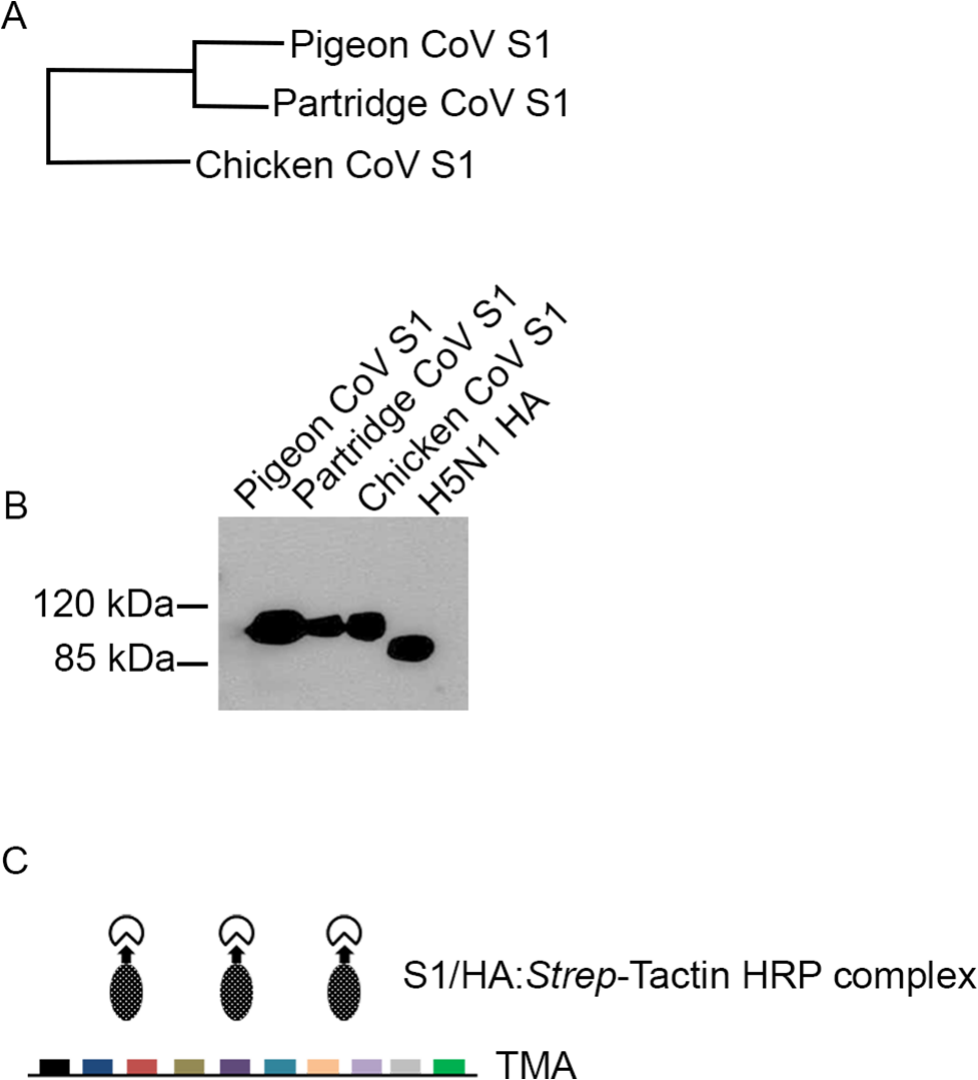
Protein expression and histochemistry. (A) Amino acid homology of pigeon CoV S1 and partridge CoV S1 to chicken CoV S1 (strain M41-S1); (B) Purified recombinant proteins analyzed by SDS-PAGE and western blotting using an antibody against the *Strep*-tag; (C) Schematic presentation of protein/spike histochemistry using TMAs.

Protein/spike histochemistry was performed as described previously [11] (schematically depicted in Fig 2C). TMA sections were rehydrated, treated with 1% hydrogen peroxide to remove endogenous peroxidases and goat serum to block nonspecific binding. Next, precomplexed S1/HA proteins (20–30 μg of S1 and 2–3 μg of HA per TMA) and *Strep*-Tactin HRP were applied to TMAs and incubated at 4°C overnight. AEC (3-amino-9-ethylcarbazole, Dako, The Netherlands) substrate was used to detect binding of proteins. To remove sialic acids, tissues were treated with *Arthrobacter ureafaciens* neuraminidase (Roche, USA) at a concentration of 1 mU/100 μl in PBS (pH 5.0) overnight at 37°Ci

### Protein histochemistry with polyacrylamide-based sialylglycopolymers

After precomplexing S1 proteins with *Strep*-Tactin HRPO for 30 min on ice either Neu5Aca2-3Galb1-3GlcNAcb-OCH2CH2CH2NH-PAA (type I lactosamine, Lectinity Holding Inc, Russia) or Neu5Aca2-3Galb1-4GlcNAcb-OCH2CH2CH2NH-PAA (type II lactosamine, Lectinity Holding Inc, Russia) were mixed in different amounts from 50 μg/ml to 200 μg/ml and incubated on ice for 30 minutes. Next these mixtures were applied to TMAs. All the other steps for protein histochemistry were as previously described [11].

### Glycan array analysis

S1 proteins of chicken CoV strain M41, pigeon CoV and partridge CoV were analyzed in the printed slides of the Consortium for Functional Glycomics (CFG) as previously described [11]. Glycan library of CFG array V5.1 was used in this experiment (Glycans included in V 5.1 are listed in S1 Table).

## Results

### Construction of TMAs to analyze tissue specific binding of viral attachment proteins

To test the potential application of TMAs in tissue-based analyses of viral attachment proteins of avian coronaviruses, tissue specimens from ten avian species from the orders *Galliformes*, *Anseriformes* and *Columbiformes* were assembled. Similar anatomical regions of respiratory tissues of the ten birds were grouped in a multi-species TMA (Fig 1A, left) to allow comparison of tissue binding patterns of viral attachment proteins of IAV and coronaviruses between different avian species. To compare host and tissue specific binding properties of various avian coronavirus spikes (S1), tissue cores from different organ systems of each bird species were grouped into single-species TMAs (Fig 1A, right). The first section from each array block was stained with H&E (Fig 1B and S1 Fig) and MALI and MALII lectins (S1 Fig) for quality control purposes. H&E staining confirmed that the tissue cores were morphologically preserved and the cores were representative specimens of the tissue archive. Lectin histochemistry using MALI and MALII confirmed the presence of sialic acids, important for the binding of S1 of coronaviruses, on the respiratory tissues from different avian species. As previously described MALII staining displayed abundance of α2,3-linked sialic acids in ciliated respiratory epithelium of chicken, turkey and goose [15], [20], [21]. While moderate to low levels of these sialic acids were observed in ciliated cells of trachea of quail, partridge, duck, guineafowl [14], [15], [21], [22] and absence or very low levels of α2,3-linked sialic acids was detected in trachea of pigeon [22]. Interestingly, MALI showed more prominent staining in trachea, while MALII staining was mostly detected in the lower respiratory tract. These staining differences between the MAL isoforms have been described previously [23].

### Binding of hemagglutinin (HA) of avian influenza virus on multi-species TMA

**Fig 3:**
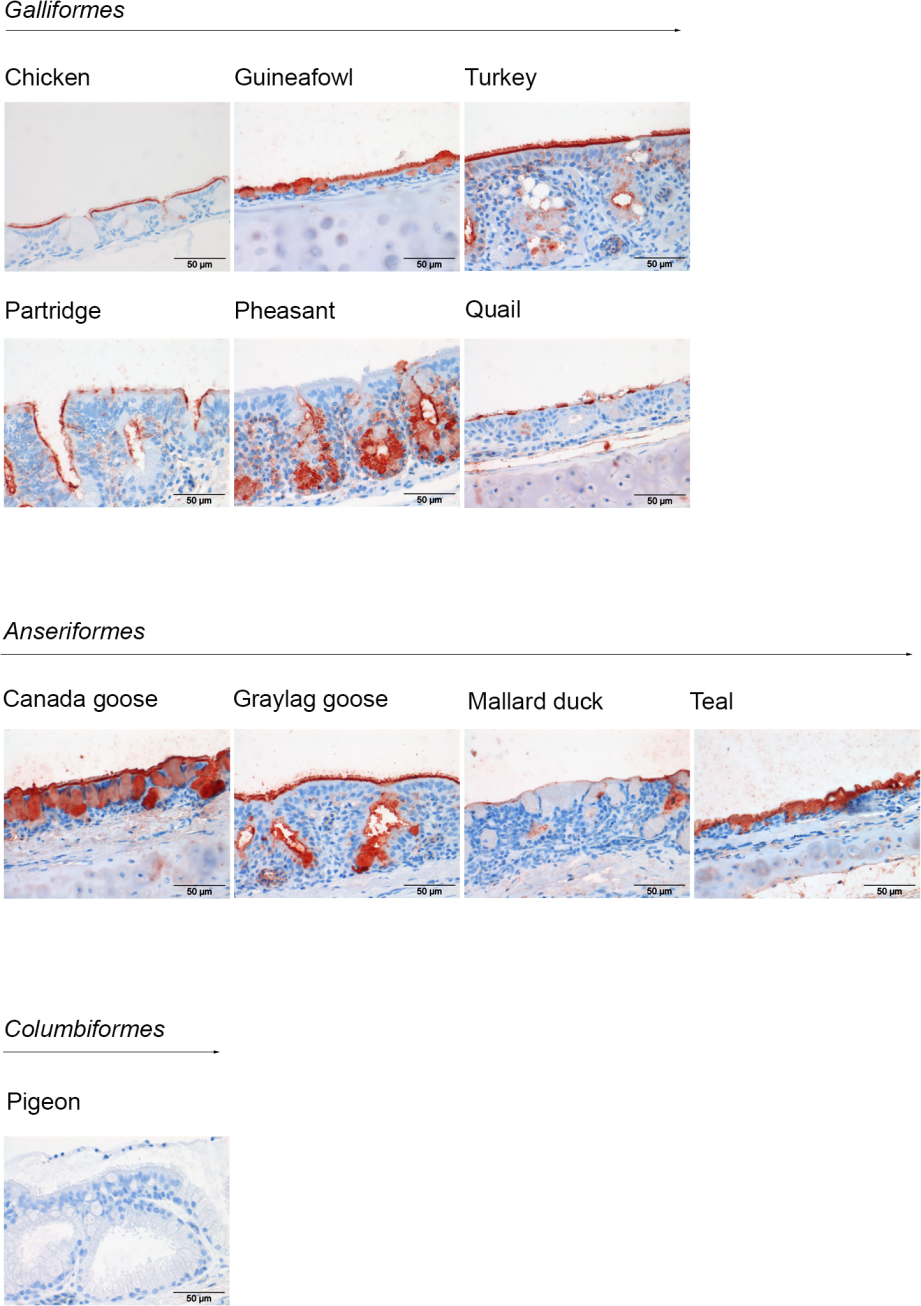
Host specificity of HA of influenza virus H5N1. HA proteins (1 μg/ml) were applied to multispecies TMA containing respiratory tissues. Binding of HA to trachea of avian species from order *Galliformes* (A), *Anseriformes* (B), and *Columbiformes* (C) is shown.

We previously showed that binding of the soluble HA of IAV H5N1 to chicken respiratory tract tissues is in agreement with the reported susceptibility of these cells for the virus [11]. To validate our multispecies TMA, we therefore first investigated whether the tissue binding profiles of HA on the respiratory tissues of the ten avian species, all well known to be susceptible for avian influenza virus [1], correlate with the preferred replication site of IAV. To this end, we expressed the ectodomain of HA of H5N1, analyzed the recombinant proteins by SDS-PAGE and western blotting (Fig 2B) and subsequently performed protein histochemistry by applying HA onto multi-species TMA (Fig 2C). A strong binding avidity for HA was detected to tracheal epithelium of chicken, guineafowl, turkey, partridge, pheasant, quail, Canada goose, graylag goose and teal (Fig 3). HA binding was observed to ciliated cells and mucous glands of the tracheal epithelium of these species. For pheasant, HA only bound to mucous glands, while it did not bind to pigeon trachea at all. Comparing to other avian species binding affinity of HA was relatively low in trachea of mallard duck. While the consequences of the differences in the avidity between species are not yet clear, it is important to note that all species showing binding of HA to the respiratory tract in our assay have been reported to be susceptible to H5N1, including chicken, guineafowl, turkey, partridge, quail, and ducks [1], [24], [25], while pigeon appeared to be resistant [22].

### Host and glycan specificity of chicken CoV S1 to multiple avian species

**Fig 4:**
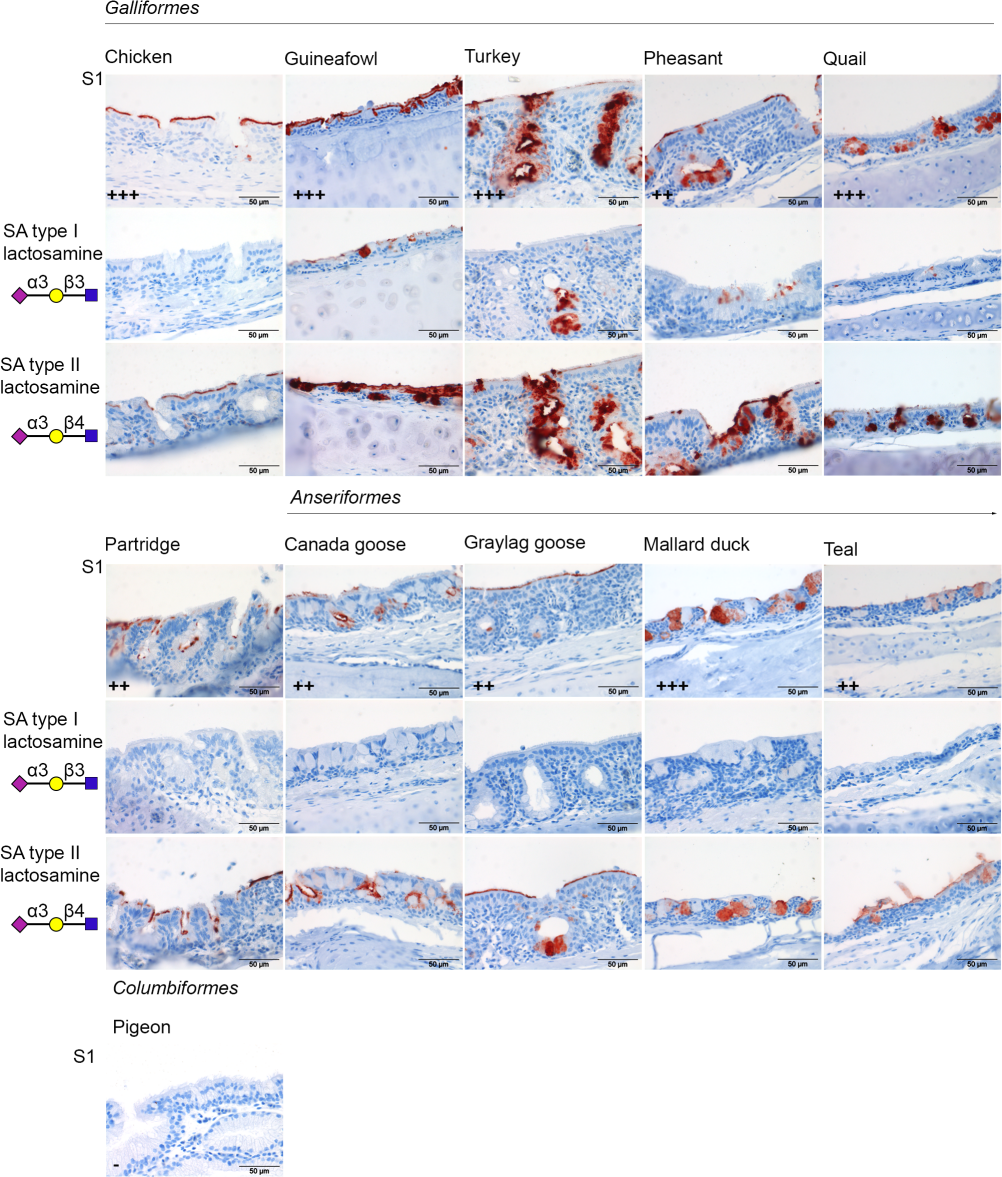
Host binding specificity of chicken CoV M41 S1. Protein histochemistry was performed by applying S1 proteins onto the trachea in the multispecies TMA. Binding affinity of chicken CoV S1 to different avian species is indicated as—(no signal), + (mild), ++ (moderate), +++ (strong). To elucidate the fine glycan specificity of S1 to sialylated glycans. S1 protein was premixed with either sialic acids (SA) type I or type II lactosamines before applying to tissues as described in materials and methods.

Next, protein histochemistry on multi-species TMA was performed using the attachment protein S1 domain of the prototype of chicken CoV M41 strain to elucidate its tissue attachment profile to respiratory tissues of various birds. We observed that chicken CoV S1 attached with higher avidity to the respiratory epithelium of the upper respiratory tract (including trachea, Fig 4, upper rows) than to the lungs of most species (Table 1). In particular, S1 attached specifically to nasal epithelium and trachea of partridge, pheasant quail, Canada goose, graylag goose, mallard duck, and teal while it only bound to the trachea, and not to the nasal epithelium of guineafowl and turkey. Interestingly, limited to no binding of chicken CoV S1 to the upper respiratory tract of pigeon was observed (Table 1). For lung, chicken CoV S1 attachment to guineafowl, teal and pigeon was observed, but not to that of other avian species (Table 1). Overall, S1 bound, as expected, only to ciliated cells and mucous glands. In particular (Fig 4), binding to the trachea was observed mainly to ciliated epithelial cells (chicken, guinea fowl and graylag goose), to the mucous glands (quail, mallard duck, teal), or both (turkey, pheasant, partridge, Canada goose). Our data indicate that an attachment factor for chicken CoV S1 is expressed on respiratory tissues of a broad range of avian species. The observed differences in signal strengths and locations between species might well reflect the abundance of the particular attachment factor, but the consequence of this for the susceptibility of the host requires further study.

**Table 1:** Binding profiles of chicken CoV S1 in respiratory tissues of various avian species.

Avian species	Nasal cavity	Trachea	Lung
Guineafowl	-	+++	+
Turkey	-	+++	-
Partridge	++	++	-
Pheasant	+++	++	-
Quail	+++	+++	-
Canada goose	+++	++	-
Graylag goose	+++	++	-
Mallard duck	++	+++	-
Teal	++	++	++
Pigeon	+	-	+++

Attachment of S1 was graded as follows:—no signal, + mild, ++moderate, +++ strong

To determine whether M41 S1 required the presence of sialic acid on the host tissues, as being a prerequisite for binding to chicken tissues [11], the multi-species TMA was treated with neuraminidase prior to applying S1 proteins. No binding of chicken CoV M41 S1 to any of the tissues of the respiratory tract multispecies TMA could be observed after neuraminidase treatment (data not shown), indicating that sialic acid is not only essential for binding to chicken tissues [11], but also to tissues of other species.

Previously, by performing glycan array analysis (CFG 4.2), we observed that M41 S1 bound to Neu5Acα2,3Galβ1,3(Neu5Acα2,3Gal1,4)-GlcNAc) [11]. Here, we first investigated the branch of the galactose that is biologically significant for binding of S1 to host tissues. To this end, synthetic sialylglycopolymer containing Neu5Acα2,3Gal1,3GlcNAc (sialic acids type I lactosamines) or Neu5Acα2,3Gal1,4GlcNAc (sialic acids type II lactosamines) were mixed with pre-complexed S1 protein and *Strep*-Tactin HRP and applied to chicken tissues. Binding of CoV S1 to the respiratory tract could be blocked with sialic acids type I at a concentration of 100 μg/ml but not with any of the concentrations (from 50 to 200 μg/ml) of type II lactosamine (Fig 4- compare second and third rows), suggesting that M41 S1 preferred Neu5Acα2,3-Gal β1,3GlcNAc over -β1,4GlcNAc subtypes on chicken tissues. In agreement with this observation, M41 S1 showed a strong binding to Neu5Acα2,3Gal1,3GlcNAc in the CFG Glycan Array V5.1 (S2 Fig) which includes both types of lactosamines.

Next, we elucidated whether binding of S1 to tissues of other avian species showed the same preference for sialic acid subtype by performing a similar blocking experiment. Sialic acids type I lactosamine could completely block the binding of S1 proteins to the trachea of *Anseriformes* (Canada goose, graylag goose, mallard duck, teal) and partridge but interestingly not that to the trachea of guineafowl, pheasant, quail, and turkey (Fig 4-second rows). For the latter, only partial blocking was observed when using 100 μg/ml lactosamines. As staining did not further reduce upon increasing the concentration of sialylglycopolymers (up to 200 μg/ml), our data suggest that the incomplete blocking was not due to higher avidity of IBV S1 to these tissues. Rather, the S1 protein may bind to different attachment factors in different avian species. Comparable to what was observed for chicken tissues sialic acids type II lactosamine could not block the binding of S1 to any of the avian species included in the multi-species TMA (Fig 4-third rows). While binding to tracheal tissues of all species depended on sialic acids, guineafowl, turkey, pheasant, and quail might allow additional binding to another yet unrevealed glycan subtype with higher avidity than the glycans bound in the natural chicken host.

### Host organ binding specificities of avian coronaviruses using single-species TMA

**Fig 5:**
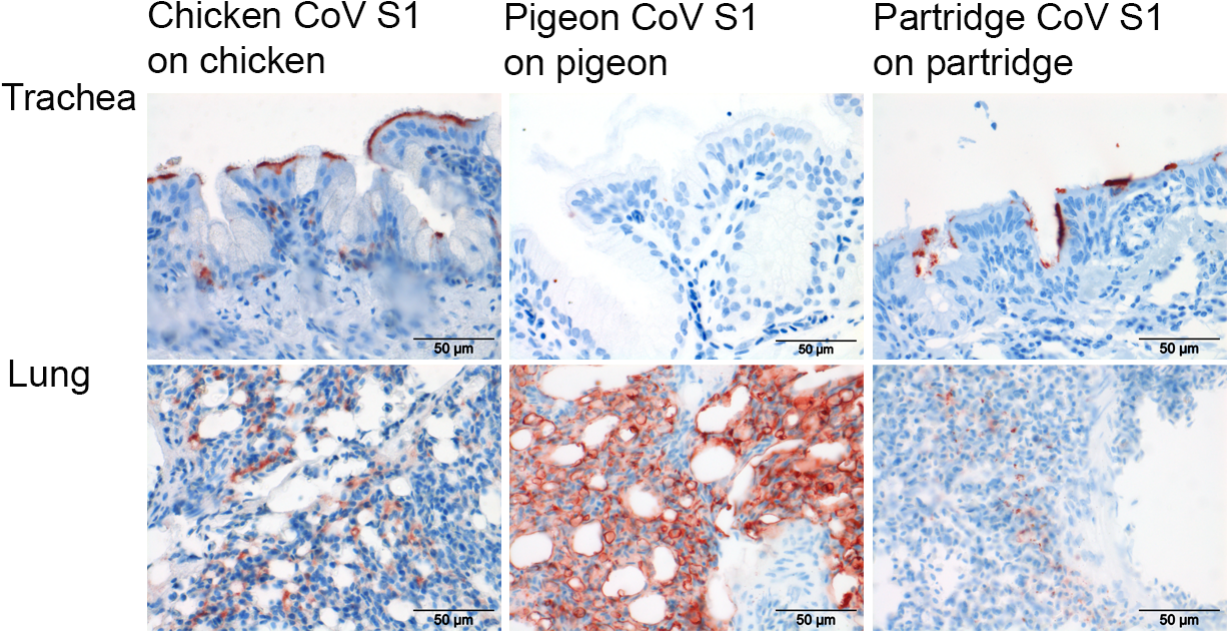
Host tissue specificity of S1 proteins of coronaviruses. Protein histochemistry was performed using chicken, pigeon and partridge CoV S1 on tissues from their respective hosts.

All chicken IBV strains show tropism primarily to the respiratory system, while some may exhibit additional preference for other organ systems, including the reproductive, urinary and gastrointestinal system [3]. We constructed the single-species TMA, containing tissues of multiple organ systems from one species, to gain more understanding on the binding preferences of a virus for particular tissues. To validate the array organ specific binding of chicken CoV S1 was profiled on single-species TMA and the binding specificities were correlated with the known tissue tropism of the chicken CoV. Chicken CoV S1 attached to trachea and lung (Fig 5), as previously observed [11]. Additionally, S1 attached to various other tissues including kidney, conjunctiva, nasal epithelium, air sacs, ileum, large intestine, and bursa of Fabricius (Table 2). Overall, the tissue attachment profiles CoV S1 was in agreement with the susceptibility of respective organ systems to chicken CoV [2].

**Table 2:** Tissue binding profiles of S1 of chicken, pigeon, and partridge CoV to the organ systems of the respective host.

Organ	Chicken CoV S1	Pigeon CoV S1	Partridge CoV S1
Conjunctiva	+++	-	++
Nasal cavity	+++	-	+++
Trachea	+++	-	+++
Lung (air capillaries)	++	+++	+
Air sacs	+	-	+++
Esophagus	-	+	+
Gizzard	-	-	-
Proventriculus	-	-	-
Duodenum	-	-	-
Ileum	++	-	+++
Large intestine	+++	++	+++
Pancrease	-	-	-
Bursa of Fabricius	++	-	-
Kidney	++	+++	++

Attachment was graded as follows:—no signal, + mild, ++moderate, +++ strong

Some gammacoronaviruses causing respiratory disease in other poultry species have high sequence homology to chicken CoV M41. In particular, the S1 domains of the pigeon and partridge CoV are 79 and 80% identical to the S1 of IBV M41 (Fig 2A). To gain insight in the role of the spike sequences in determining the host tropism and to elucidate the presence of attachment factors on avian species for those CoV, we expressed recombinant S1 of pigeon and partridge CoV. As expected, migration patterns of S1 proteins of pigeon and partridge CoVs were comparable to that of IBV M41 S1 (molecular weights of approximately 110 kDa (Fig 2B). To determine the fine glycan specificity of pigeon and partridge CoV S1, the S1 proteins were first analyzed by glycan array analysis. Unfortunately, S1 of pigeon and partridge CoV did not recognize any of the 610 specific glycans present in the glycan array V5.1 of the CFG (data not shown).

**Fig 6:**
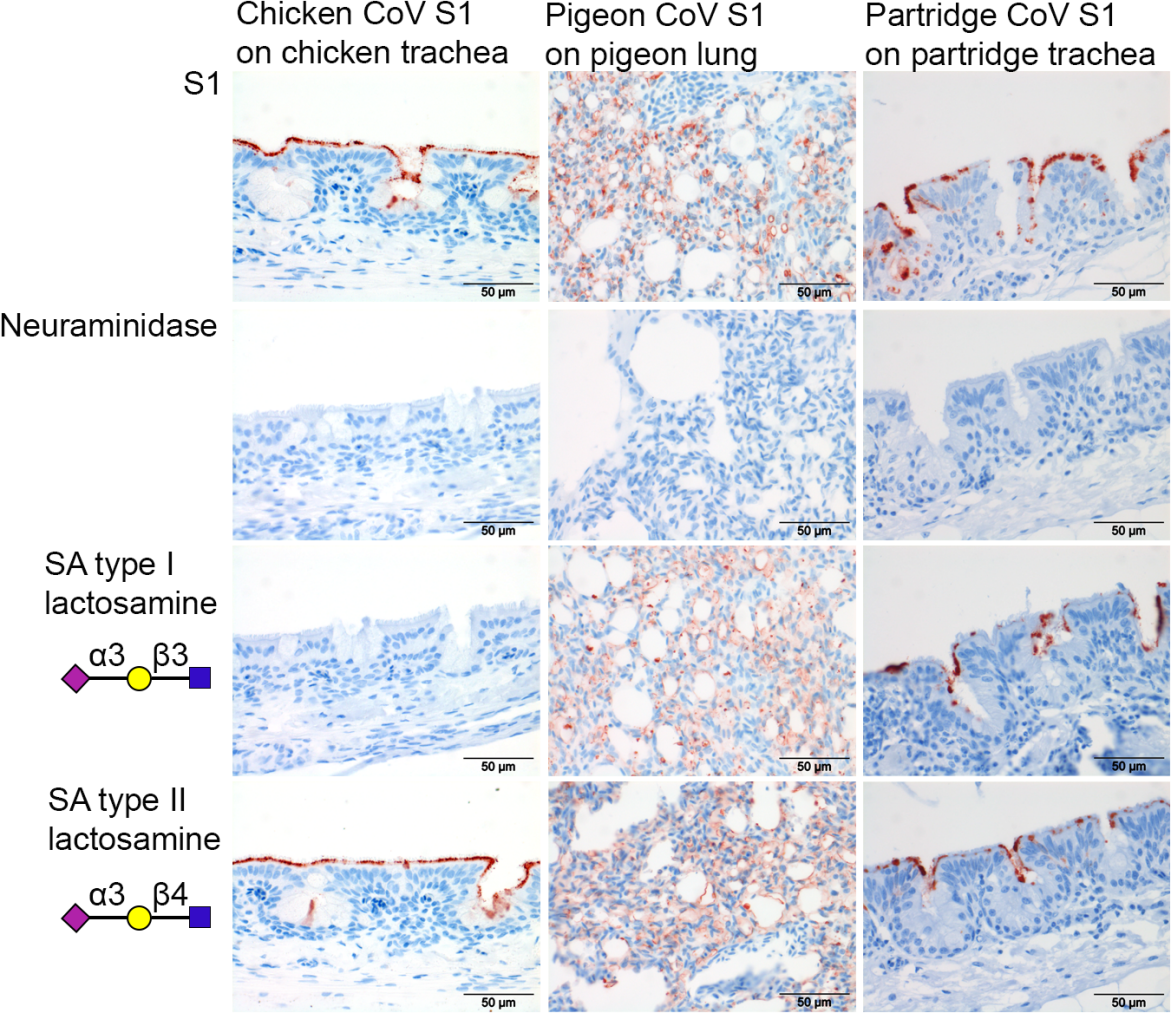
Glycan binding specificities of pigeon, partridge and chicken CoV S1. Before applying S1, TMAs were either treated non treated (S1) or pretreated with neuraminidase (NA), or S1 proteins were mixed with sialic acids (SA) type I or type II lactosamines.

The single species TMA of pigeon and partridge was next used to elucidate binding characteristics of these proteins to their respective hosts. When applied to pigeon tissues, pigeon CoV S1 showed strong attachment to the lung and only mild binding to other pigeon tissues (Fig 5 & Table 2). In contrast, partridge CoV S1 showed not only strong attachment to the respiratory tract but also to intestinal tissues of partridge (Fig 5 & Table 2). These data suggest that pigeon CoV S1 and partridge CoV S1 possess, as chicken CoV M41 S1, high affinity to respiratory system of the particular host in which the virus was detected. There are, however, marked differences in the avidity and specificity of each of the spike proteins for tissues of their respective hosts. To further investigate this, we studied the contribution of host sialic acids in the binding of pigeon CoV S1 and partridge CoV S1. Therefore, we first applied the S1 proteins onto our single-species TMA pretreated with neuraminidase. Binding of pigeon, partridge and chicken S1 proteins to the respective TMA was completely abolished (Fig 6, second row), suggesting that all studied S1 proteins required sialic acid for tissue attachment. However, mixing the S1 proteins with either sialic acids type I lactosamine and type II lactosamine showed that the binding of pigeon CoV S1 and partridge CoV S1 could not be blocked by any of these specific glycans (Fig 6-third and fourth row), in contrast to the observed lack of binding of M41 S1 in the presence of type I lactosamine. In conclusion, these results suggest that although tissue binding of pigeon CoV-S1 and partridge CoV-S1 is sialic acid dependent, the specific α2,3-linked sialic acid prerequisites for binding to tissues are not identical to that of chicken CoV M41S1. As both of these S1 proteins did not recognize any specific glycans in the glycan array the fine glycan specificity of these S1 proteins remains to be elucidated.

## Discussion

In this study we demonstrate that avian tissue microarrays are an excellent platform to elucidate host and tissue binding specificities of viral attachment proteins. In particular, this study revealed that the chicken coronavirus might have a broader host tropism than previously thought, as the IBV spike protein could bind to the respiratory epithelium of various avian species. Interestingly, this appeared to be due to differences in fine glycan specificity, although binding to the respiratory tract of other birds was still sialic acid dependent. The IBV spike tissue binding affinity was, however, markedly less compared to that of the H5 hemagglutinin of IAV H5N1, a well-known avian pathogen with tropism for many avian species. S1 proteins of IBV-like coronaviruses, including that of pigeon and partridge, also showed primarily tropism to the respiratory system of their respective host. Despite that sialic acid dependent binding was retained, however, their fine glycan specificity appeared to differ between avian coronaviruses identified in different hosts.

Our data show that the S1 domain of IBV M41 can attach to respiratory tissues of birds other than chicken, including that of Canada goose, graylag goose, guineafowl, mallard duck, partridge, pheasant, quail, teal and turkey. These data indicate that these birds express the first, but important host attachment factor for this virus. Therefore, the binding spectrum of S1 currently suggests that these avian species may be susceptible for chicken CoV. Coronaviruses genetically close to chicken CoV have been detected in various other avian species including both domestic and wild birds [2], [26–28] indicating that IBV or IBV-like viruses circulate in birds and that these viruses might infect more than one avian species [2], [28]. It is not yet clear whether the observed difference in the binding affinity of S1, from mild to strong, to tissues can be correlated with the degree of pathogenicity or tropism of the virus to a particular tissue. Previously, we have shown that the binding affinity of recombinant S1 proteins of chicken CoV Massachusetts strains to chicken respiratory tract correlated to the reported pathogenicity of these viruses [11]. It might well be that the presence of sialic acids on tissues in part define the susceptibility of tissues, while other viral [10] and host factors contribute to the pathogenicity additionally. Further conclusions on the actual susceptibility of these avian species for chicken CoV and pathogenicity of this virus in these avian species will require *in vivo* infections studies.

To elucidate specific host attachment factors, and to distinguish between glycan specificities of attachment proteins, the combined use of neuraminidases and specific sialylglycopolymers in tissue-based approaches can be employed. Here we showed that chicken CoV S1 prefers α2,3-linked sialic acids type I lactosamine over type II lactosamine on chicken tissues. Additionally, our data reveal that binding of chicken CoV to various other avian species, while depending on sialic acids, did not require this particular sialylated lactosamine. Previously, preferential binding of influenza viruses originating from different avian species has been described to differ in their preference for specific sialylated glycans [20], [29]. While influenza viruses from duck preferred α2,3-linked sialic acids type I lactosamine, IAV from chicken preferred type II lactosamine [20], [30] indicating that elucidation of the distribution of specific sialic acid sub types can provide insight into susceptibility for a particular pathogen. The lack of the ability to block chicken CoV S1 binding to guineafowl, pheasant, quail and turkey with type I lactosamine may suggest that IBV S1 recognizes additional sialylated glycans which may not be included in the glycan microarray [31]. Similarly, S1 proteins of pigeon and partridge CoV showed marked differences in their affinities to tissues of their respective host, albeit of no specific glycan could yet be discovered in the glycan array (data not shown). Despite this, our current data suggest that the fine glycan specificity of pigeon and partridge CoV S1 is different from that of the chicken IBV M41.

Chicken CoV strain IBV M41 infects primarily tissues of the respiratory system, but also other organs including kidney and intestine [3]. On our single-species TMA, binding was detected to multiple organs, not only to the kidney and intestine (this paper, [11]), but also to the conjunctiva, bursa of Fabricius (this paper), and oviduct (data not shown). The binding profile was comparable to the reported *in vivo* tissue tropism of IBV M41 in chickens [2], [3][2], [26–28]. As for IBV-like viruses, including that of pigeon [2], [6] and partridge [2], hardly anything is known about the tissue tropism, our tissue binding profiles provide the first insight on the preference of these viruses for organ systems. Interestingly, binding of pigeon CoV S1 to pigeon tissues was limited to lung and intestine. In this respect, it is of interest to mention the limited susceptibility of pigeon for IAV, which has been associated with the lack of α 2,3-linked sialic acids in the upper respiratory tract [22]. Likely, this differential expression of host glycans might have caused the marked difference in binding of pigeon CoV as well. Partridge CoV S1 appeared to have a more comparable preference for host factors as IBV S1, as mainly binding to the respiratory and intestinal systems of partridge was observed. Although these initial valuable insights into the interactions between avian gammacoronaviruses and potential hosts, more data is required to ultimately conclude on the susceptibility of these birds for coronaviruses.

We have shown that TMAs are an efficient platform to analyze host and organ preferences of viral attachment proteins on multiple tissues at the same time. This tissue-based method is in fact an excellent method to assess the first step in the virus infection cycle for viruses for which limited model systems are available. TMAs were first introduced into the field of oncology for rapid and cost effective screening of molecular markers [32]. Serial sections from TMA accelerated the screening of hundreds of molecular markers targeted at RNA, DNA or proteins on the same tissue at the same time while reproducing similar data sets that one could obtained from individual sections [32], [33]. Here we demonstrated that in the field of virology by grouping 50–60 tissue samples in one microscopic slide TMA enabled rapid detection of tissue specific binding properties of S1 proteins of various avian coronaviruses including that of chicken, pigeon, and partridge. Our current avian TMA required only 20–30 μg of recombinant proteins, which is approximately 20 times less recombinant protein than that would be required for individual tissue sections. By developing arrays as multi-species and single-species tissue arrays, binding specificities of S1 proteins could easily be compared between various avian species and between different organ systems.

In conclusion, tissue microarrays provide a fast and cost effective way to elucidate host and tissue binding profiles of viral attachment proteins. Such assays can provide novel information on the tropism of avian viruses including specific host factors involved. Further, tissue-binding specificities revealed that chicken CoV might have a broader species tropism than previously believed and specific sialylated glycans might actually contribute to the diverse outcome of the various avian gammacoronaviruses.

## Supporting Information

S1 Table Glycan library of CFG array version 5.1. (PDF)

S1 Fig TMA quality control using H&E and lectins. TMAs were stained with H&E (first rows) to confirm that tissue cores were morphologically preserved and the cores were representative specimens of the tissue archive. TMAs were stained with lectins (MALI/second rows and MALII/ third rows) to confirm the presence of sialic acids.
(TIF)

S2 Fig Glycan array analysis of chicken CoV strain M41 S1. Printed slides contained the glycan library of CFG array version 5.1
(TIF)
