# Supplementary material for: Host Tissue and Glycan Binding Specificities of Avian Viral Attachment Proteins Using Novel Avian Tissue Microarrays
Source: PLoS One. 2015 Jun 2;10(6):e0128893. doi: 10.1371/journal.pone.0128893 (PMC4452732; doi:10.1371/journal.pone.0128893)
Supplement: S1 Table — (PDF) [file pone.0128893.s001.pdf]

Version 5.1

Chart Number	Structure on Masterlist
1	Gala-Sp8
2	Glca-Sp8
3	Mana-Sp8
4	GalNAca-Sp8
5	GalNAca-Sp15
6	Fuca-Sp8
7	Fuca-Sp9
8	Rhaa-Sp8
9	Neu5Aca-Sp8
10	Neu5Aca-Sp11
11	Neu5Acb-Sp8
12	Galb-Sp8
13	Glcb-Sp8
14	Manb-Sp8
15	GalNAcb-Sp8
16	GlcNAcb-Sp0
17	GlcNAcb-Sp8
18	GlcN(Gc)b-Sp8
19	Galb1-4GlcNAcb1-6(Galb1-4GlcNAcb1-3)GalNAca-Sp8
20	Galb1-4GlcNAcb1-6(Galb1-4GlcNAcb1-3)GalNAc-Sp14
21	GlcNAcb1-6(GlcNAcb1-4)(GlcNAcb1-3)GlcNAc-Sp8
22	6S(3S)Galb1-4(6S)GlcNAcb-Sp0
23	6S(3S)Galb1-4GlcNAcb-Sp0
24	(3S)Galb1-4(Fuca1-3)(6S)Glc-Sp0
25	(3S)Galb1-4Glcb-Sp8
26	(3S)Galb1-4(6S)Glcb-Sp0
27	(3S)Galb1-4(6S)Glcb-Sp8
28	(3S)Galb1-3(Fuca1-4)GlcNAcb-Sp8
29	(3S)Galb1-3GalNAca-Sp8
30	(3S)Galb1-3GlcNAcb-Sp0
31	(3S)Galb1-3GlcNAcb-Sp8
32	(3S)Galb1-4(Fuca1-3)GlcNAc-Sp0
33	(3S)Galb1-4(Fuca1-3)GlcNAc-Sp8
34	(3S)Galb1-4(6S)GlcNAcb-Sp0
35	(3S)Galb1-4(6S)GlcNAcb-Sp8
36	(3S)Galb1-4GlcNAcb-Sp0
37	(3S)Galb1-4GlcNAcb-Sp8
38	(3S)Galb-Sp8
39	(6S)(4S)Galb1-4GlcNAcb-Sp0
40	(4S)Galb1-4GlcNAcb-Sp8
41	(6P)Mana-Sp8
42	(6S)Galb1-4Glcb-Sp0
43	(6S)Galb1-4Glcb-Sp8
44	(6S)Galb1-4GlcNAcb-Sp8
45	(6S)Galb1-4(6S)Glcb-Sp8
46	Neu5Aca2-3(6S)Galb1-4GlcNAcb-Sp8
47	(6S)GlcNAcb-Sp8
48	Neu5,9Ac$_2$a-Sp8
49	Neu5,9Ac2a2-6Galb1-4GlcNAcb-Sp8
50	Mana1-6(Mana1-3)Manb1-4GlcNAcb1-4GlcNAcb-Sp12
51	Mana1-6(Mana1-3)Manb1-4GlcNAcb1-4GlcNAcb-Sp13
52	GlcNAcb1-2Mana1-6(GlcNAcb1-2Mana1-3)Manb1-4GlcNAcb1-4GlcNAcb-Sp12
53	GlcNAcb1-2Mana1-6(GlcNAcb1-2Mana1-3)Manb1-4GlcNAcb1-4GlcNAcb-Sp13
54	Galb1-4GlcNAcb1-2Mana1-6(Galb1-4GlcNAcb1-2Mana1-3)Manb1-4GlcNAcb1-4GlcNAcb-Sp12
55	Neu5Aca2-6Galb1-4GlcNAcb1-2Mana1-6(Neu5Aca2-6Galb1-4GlcNAcb1-2Mana1-3)Manb1-4GlcNAcb1-4GlcNAcb-Sp12
56	Neu5Aca2-6Galb1-4GlcNAcb1-2Mana1-6(Neu5Aca2-6Galb1-4GlcNAcb1-2Man-a1-3)Manb1-4GlcNAcb1-4GlcNAcb-Sp21
57	Neu5Aca2-6Galb1-4GlcNAcb1-2Mana1-6(Neu5Aca2-6Galb1-4GlcNAcb1-2Mana1-3)Manb1-4GlcNAcb1-4GlcNAcb-Sp24
58	Fuca1-2Galb1-3GalNAcb1-3Gala-Sp9

Chart Number	Structure on Masterlist
59	Fuca1-2Galb1-3GalNAcb1-3Gala1-4Galb1-4Glcb-Sp9
60	Fuca1-2Galb1-3(Fuca1-4)GlcNAcb-Sp8
61	Fuca1-2Galb1-3GalNAca-Sp8
62	Fuca1-2Galb1-3GalNAca-Sp14
63	Fuca1-2Galb1-3GalNAcb1-4(Neu5Aca2-3)Galb1-4Glcb-Sp0
64	Fuca1-2Galb1-3GalNAcb1-4(Neu5Aca2-3)Galb1-4Glcb-Sp9
65	Fuca1-2Galb1-3GlcNAcb1-3Galb1-4Glcb-Sp8
66	Fuca1-2Galb1-3GlcNAcb1-3Galb1-4Glcb-Sp10
67	Fuca1-2Galb1-3GlcNAcb-Sp0
68	Fuca1-2Galb1-3GlcNAcb-Sp8
69	Fuca1-2Galb1-4(Fuca1-3)GlcNAcb1-3Galb1-4(Fuca1-3)GlcNAcb-Sp0
70	Fuca1-2Galb1-4(Fuca1-3)GlcNAcb1-3Galb1-4(Fuca1-3)GlcNAcb1-3Galb1-4(Fuca1-3)GlcNAcb-Sp0
71	Fuca1-2Galb1-4(Fuca1-3)GlcNAcb-Sp0
72	Fuca1-2Galb1-4(Fuca1-3)GlcNAcb-Sp8
73	Fuca1-2Galb1-4GlcNAcb1-3Galb1-4GlcNAcb-Sp0
74	Fuca1-2Galb1-4GlcNAcb1-3Galb1-4GlcNAcb1-3Galb1-4GlcNAcb-Sp0
75	Fuca1-2Galb1-4GlcNAcb-Sp0
76	Fuca1-2Galb1-4GlcNAcb-Sp8
77	Fuca1-2Galb1-4Glcb-Sp0
78	Fuca1-2Galb-Sp8
79	Fuca1-3GlcNAcb-Sp8
80	Fuca1-4GlcNAcb-Sp8
81	Fucb1-3GlcNAcb-Sp8
82	GalNAca1-3(Fuca1-2)Galb1-3GlcNAcb-Sp0
83	GalNAca1-3(Fuca1-2)Galb1-4(Fuca1-3)GlcNAcb-Sp0
84	(3S)Galb1-4(Fuca1-3)Glcb-Sp0
85	GalNAca1-3(Fuca1-2)Galb1-4GlcNAcb-Sp0
86	GalNAca1-3(Fuca1-2)Galb1-4GlcNAcb-Sp8
87	GalNAca1-3(Fuca1-2)Galb1-4Glcb-Sp0
88	GlcNAcb1-3Galb1-3GalNAca-Sp8
89	GalNAca1-3(Fuca1-2)Galb-Sp8
90	GalNAca1-3(Fuca1-2)Galb-Sp18
91	GalNAca1-3GalNAcb-Sp8
92	GalNAca1-3Galb-Sp8
93	GalNAca1-4(Fuca1-2)Galb1-4GlcNAcb-Sp8
94	GalNAcb1-3GalNAca-Sp8
95	GalNAcb1-3(Fuca1-2)Galb-Sp8
96	GalNAcb1-3Gala1-4Galb1-4GlcNAcb-Sp0
97	GalNAcb1-4(Fuca1-3)GlcNAcb-Sp0
98	GalNAcb1-4GlcNAcb-Sp0
99	GalNAcb1-4GlcNAcb-Sp8
100	Gala1-2Galb-Sp8
101	Gala1-3(Fuca1-2)Galb1-3GlcNAcb-Sp0
102	Gala1-3(Fuca1-2)Galb1-3GlcNAcb-Sp8
103	Gala1-3(Fuca1-2)Galb1-4(Fuca1-3)GlcNAcb-Sp0
104	Gala1-3(Fuca1-2)Galb1-4(Fuca1-3)GlcNAcb-Sp8
105	Gala1-3(Fuca1-2)Galb1-4GlcNAc-Sp0
106	Gala1-3(Fuca1-2)Galb1-4Glcb-Sp0
107	Gala1-3(Fuca1-2)Galb-Sp8
108	Gala1-3(Fuca1-2)Galb-Sp18
109	Gala1-4(Gala1-3)Galb1-4GlcNAcb-Sp8
110	Gala1-3GalNAca-Sp8
111	Gala1-3GalNAca-Sp16
112	Gala1-3GalNAcb-Sp8
113	Gala1-3Galb1-4(Fuca1-3)GlcNAcb-Sp8
114	Gala1-3Galb1-3GlcNAcb-Sp0
115	Gala1-3Galb1-4GlcNAcb-Sp8
116	Gala1-3Galb1-4Glcb-Sp0
117	Gala1-3Galb1-4Glc-Sp10
118	Gala1-3Galb-Sp8
119	Gala1-4(Fuca1-2)Galb1-4GlcNAcb-Sp8
120	Gala1-4Galb1-4GlcNAcb-Sp0
121	Gala1-4Galb1-4GlcNAcb-Sp8

Chart Number	Structure on Masterlist
122	Gala1-4Galb1-4Glcb-Sp0
123	Gala1-4GlcNAcb-Sp8
124	Gala1-6Glcb-Sp8
125	Galb1-2Galb-Sp8
126	Galb1-3(Fuca1-4)GlcNAcb1-3Galb1-4(Fuca1-3)GlcNAcb-Sp0
127	Galb1-3GlcNAcb1-3Galb1-4(Fuca1-3)GlcNAcb-Sp0
128	Galb1-3(Fuca1-4)GlcNAc-Sp0
129	Galb1-3(Fuca1-4)GlcNAc-Sp8
130	Fuca1-4(Galb1-3)GlcNAcb-Sp8
131	Galb1-4GlcNAcb1-6GalNAca-Sp8
132	Galb1-4GlcNAcb1-6GalNAc-Sp14
133	GlcNAcb1-6(Galb1-3)GalNAca-Sp8
134	GlcNAcb1-6(Galb1-3)GalNAca-Sp14
135	Neu5Aca2-6(Galb1-3)GalNAca-Sp8
136	Neu5Aca2-6(Galb1-3)GalNAca-Sp14
137	Neu5Acb2-6(Galb1-3)GalNAca-Sp8
138	Neu5Aca2-6(Galb1-3)GlcNAcb1-4Galb1-4Glcb-Sp10
139	Galb1-3GalNAca-Sp8
140	Galb1-3GalNAca-Sp14
141	Galb1-3GalNAca-Sp16
142	Galb1-3GalNAcb-Sp8
143	Galb1-3GalNAcb1-3Gala1-4Galb1-4Glcb-Sp0
144	Galb1-3GalNAcb1-4(Neu5Aca2-3)Galb1-4Glcb-Sp0
145	Galb1-3GalNAcb1-4Galb1-4Glcb-Sp8
146	Galb1-3Galb-Sp8
147	Galb1-3GlcNAcb1-3Galb1-4GlcNAcb-Sp0
148	Galb1-3GlcNAcb1-3Galb1-4Glcb-Sp10
149	Galb1-3GlcNAcb-Sp0
150	Galb1-3GlcNAcb-Sp8
151	Galb1-4(Fuca1-3)GlcNAcb-Sp0
152	Galb1-4(Fuca1-3)GlcNAcb-Sp8
153	Galb1-4(Fuca1-3)GlcNAcb1-3Galb1-4(Fuca1-3)GlcNAcb-Sp0
154	Galb1-4(Fuca1-3)GlcNAcb1-3Galb1-4(Fuca1-3)GlcNAcb1-3Galb1-4(Fuca1-3)GlcNAcb-Sp0
155	Galb1-4(6S)Glcb-Sp0
156	Galb1-4(6S)Glcb-Sp8
157	Galb1-4GalNAca1-3(Fuca1-2)Galb1-4GlcNAcb-Sp8
158	Galb1-4GalNAcb1-3(Fuca1-2)Galb1-4GlcNAcb-Sp8
159	Galb1-4GlcNAcb1-3GalNAca-Sp8
160	Galb1-4GlcNAcb1-3GalNAc-Sp14
161	Galb1-4GlcNAcb1-3Galb1-4(Fuca1-3)GlcNAcb1-3Galb1-4(Fuca1-3)GlcNAcb-Sp0
162	Galb1-4GlcNAcb1-3Galb1-4GlcNAcb1-3Galb1-4GlcNAcb-Sp0
163	Galb1-4GlcNAcb1-3Galb1-4GlcNAcb-Sp0
164	Galb1-4GlcNAcb1-3Galb1-4Glcb-Sp0
165	Galb1-4GlcNAcb1-3Galb1-4Glcb-Sp8
166	Galb1-4GlcNAcb1-6(Galb1-3)GalNAca-Sp8
167	Galb1-4GlcNAcb1-6(Galb1-3)GalNAc-Sp14
168	Galb1-4GlcNAcb-Sp0
169	Galb1-4GlcNAcb-Sp8
170	Galb1-4GlcNAcb-Sp23
171	Galb1-4Glcb-Sp0
172	Galb1-4Glcb-Sp8
173	GlcNAca1-3Galb1-4GlcNAcb-Sp8
174	GlcNAca1-6Galb1-4GlcNAcb-Sp8
175	GlcNAcb1-2Galb1-3GalNAca-Sp8
176	GlcNAcb1-6(GlcNAcb1-3)GalNAca-Sp8
177	GlcNAcb1-6(GlcNAcb1-3)GalNAca-Sp14
178	GlcNAcb1-6(GlcNAcb1-3)Galb1-4GlcNAcb-Sp8
179	GlcNAcb1-3GalNAca-Sp8
180	GlcNAcb1-3GalNAca-Sp14
181	GlcNAcb1-3Galb-Sp8
182	GlcNAcb1-3Galb1-4GlcNAcb-Sp0
183	GlcNAcb1-3Galb1-4GlcNAcb-Sp8
184	GlcNAcb1-3Galb1-4GlcNAcb1-3Galb1-4GlcNAcb-Sp0

Chart Number	Structure on Masterlist
185	GlcNAcb1-3Galb1-4Glcb-Sp0
186	GlcNAcb1-4-MDPLys
187	GlcNAcb1-6(GlcNAcb1-4)GalNAca-Sp8
188	GlcNAcb1-4Galb1-4GlcNAcb-Sp8
189	GlcNAcb1-4GlcNAcb1-4GlcNAcb1-4GlcNAcb1-4GlcNAcb1-4GlcNAcb1-Sp8
190	GlcNAcb1-4GlcNAcb1-4GlcNAcb1-4GlcNAcb1-4GlcNAcb1-Sp8
191	GlcNAcb1-4GlcNAcb1-4GlcNAcb-Sp8
192	GlcNAcb1-6GalNAca-Sp8
193	GlcNAcb1-6GalNAca-Sp14
194	GlcNAcb1-6Galb1-4GlcNAcb-Sp8
195	Glca1-4Glcb-Sp8
196	Glca1-4Glca-Sp8
197	Glca1-6Glca1-6Glcb-Sp8
198	Glcb1-4Glcb-Sp8
199	Glcb1-6Glcb-Sp8
200	G-ol-Sp8
201	GlcAa-Sp8
202	GlcAb-Sp8
203	GlcAb1-3Galb-Sp8
204	GlcAb1-6Galb-Sp8
205	KDNa2-3Galb1-3GlcNAcb-Sp0
206	KDNa2-3Galb1-4GlcNAcb-Sp0
207	Mana1-2Mana1-2Mana1-3Mana-Sp9
208	Mana1-2Mana1-6(Mana1-2Mana1-3)Mana-Sp9
209	Mana1-2Mana1-3Mana-Sp9
210	Mana1-6(Mana1-2Mana1-3)Mana1-6(Mana1-2Mana1-3)Manb1-4GlcNAcb1-4GlcNAcb-Sp12
211	Mana1-2Mana1-6(Mana1-3)Mana1-6(Mana1-2Mana1-2Mana1-3)Manb1-4GlcNAcb1-4GlcNAcb-Sp12
212	Mana1-2Mana1-6(Mana1-2Mana1-3)Mana1-6(Mana1-2Mana1-2Mana1-3)Manb1-4GlcNAcb1-4GlcNAcb-Sp12
213	Mana1-6(Mana1-3)Mana-Sp9
214	Mana1-2Mana1-2Mana1-6(Mana1-3)Mana-Sp9
215	Mana1-6(Mana1-3)Mana1-6(Mana1-2Mana1-3)Manb1-4GlcNAcb1-4GlcNAcb-Sp12
216	Mana1-6(Mana1-3)Mana1-6(Mana1-3)Manb1-4GlcNAcb1-4GlcNAcb-Sp12
217	Manb1-4GlcNAcb-Sp0
218	Neu5Aca2-3Galb1-4GlcNAcb1-3Galb1-4(Fuca1-3)GlcNAcb-Sp0
219	(3S)Galb1-4(Fuca1-3)(6S)GlcNAcb-Sp8
220	Fuca1-2(6S)Galb1-4GlcNAcb-Sp0
221	Fuca1-2Galb1-4(6S)GlcNAcb-Sp8
222	Fuca1-2(6S)Galb1-4(6S)Glcb-Sp0
223	Neu5Aca2-3Galb1-3GalNAca-Sp8
224	Neu5Aca2-3Galb1-3GalNAca-Sp14
225	GalNAcb1-4(Neu5Aca2-8Neu5Aca2-8Neu5Aca2-8Neu5Aca2-3)Galb1-4Glcb-Sp0
226	GalNAcb1-4(Neu5Aca2-8Neu5Aca2-8Neu5Aca2-3)Galb1-4Glcb-Sp0
227	Neu5Aca2-8Neu5Aca2-8Neu5Aca2-3Galb1-4Glcb-Sp0
228	GalNAcb1-4(Neu5Aca2-8Neu5Aca2-3)Galb1-4Glcb-Sp0
229	Neu5Aca2-8Neu5Aca2-8Neu5Aca-Sp8
230	Neu5Aca2-3(6S)Galb1-4(Fuca1-3)GlcNAcb-Sp8
231	GalNAcb1-4(Neu5Aca2-3)Galb1-4GlcNAcb-Sp0
232	GalNAcb1-4(Neu5Aca2-3)Galb1-4GlcNAcb-Sp8
233	GalNAcb1-4(Neu5Aca2-3)Galb1-4Glcb-Sp0
234	Neu5Aca2-3Galb1-3GalNAcb1-4(Neu5Aca2-3)Galb1-4Glcb-Sp0
235	Neu5Aca2-6(Neu5Aca2-3)GalNAca-Sp8
236	Neu5Aca2-3GalNAca-Sp8
237	Neu5Aca2-3GalNAcb1-4GlcNAcb-Sp0
238	Neu5Aca2-3Galb1-3(6S)GlcNAc-Sp8
239	Neu5Aca2-3Galb1-3(Fuca1-4)GlcNAcb-Sp8
240	Neu5Aca2-3Galb1-3(Fuca1-4)GlcNAcb1-3Galb1-4(Fuca1-3)GlcNAcb-Sp0
241	Neu5Aca2-3Galb1-4(Neu5Aca2-3Galb1-3)GlcNAcb-Sp8
242	Neu5Aca2-3Galb1-3(6S)GalNAca-Sp8
243	Neu5Aca2-6(Neu5Aca2-3Galb1-3)GalNAca-Sp8
244	Neu5Aca2-6(Neu5Aca2-3Galb1-3)GalNAca-Sp14
245	Neu5Aca2-3Galb-Sp8
246	Neu5Aca2-3Galb1-3GalNAcb1-3Gala1-4Galb1-4Glcb-Sp0
247	Neu5Aca2-3Galb1-3GlcNAcb1-3Galb1-4GlcNAcb-Sp0

Chart Number	Structure on Masterlist
248	Fuca1-2(6S)Galb1-4Glcb-Sp0
249	Neu5Aca2-3Galb1-3GlcNAcb-Sp0
250	Neu5Aca2-3Galb1-3GlcNAcb-Sp8
251	Neu5Aca2-3Galb1-4(6S)GlcNAcb-Sp8
252	Neu5Aca2-3Galb1-4(Fuca1-3)(6S)GlcNAcb-Sp8
253	Neu5Aca2-3Galb1-4(Fuca1-3)GlcNAcb1-3Galb1-4(Fuca1-3)GlcNAcb1-3Galb1-4(Fuca1-3)GlcNAcb-Sp0
254	Neu5Aca2-3Galb1-4(Fuca1-3)GlcNAcb-Sp0
255	Neu5Aca2-3Galb1-4(Fuca1-3)GlcNAcb-Sp8
256	Neu5Aca2-3Galb1-4(Fuca1-3)GlcNAcb1-3Galb-Sp8
257	Neu5Aca2-3Galb1-4(Fuca1-3)GlcNAcb1-3Galb1-4GlcNAcb-Sp8
258	Neu5Aca2-3Galb1-4GlcNAcb1-3Galb1-4GlcNAcb1-3Galb1-4GlcNAcb-Sp0
259	Neu5Aca2-3Galb1-4GlcNAcb-Sp0
260	Neu5Aca2-3Galb1-4GlcNAcb-Sp8
261	Neu5Aca2-3Galb1-4GlcNAcb1-3Galb1-4GlcNAcb-Sp0
262	Fuca1-2Galb1-4(6S)Glcb-Sp0
263	Neu5Aca2-3Galb1-4Glcb-Sp0
264	Neu5Aca2-3Galb1-4Glcb-Sp8
265	Neu5Aca2-6GalNAca-Sp8
266	Neu5Aca2-6GalNAcb1-4GlcNAcb-Sp0
267	Neu5Aca2-6Galb1-4(6S)GlcNAcb-Sp8
268	Neu5Aca2-6Galb1-4GlcNAcb-Sp0
269	Neu5Aca2-6Galb1-4GlcNAcb-Sp8
270	Neu5Aca2-6Galb1-4GlcNAcb1-3Galb1-4(Fuca1-3)GlcNAcb1-3Galb1-4(Fuca1-3)GlcNAcb-Sp0
271	Neu5Aca2-6Galb1-4GlcNAcb1-3Galb1-4GlcNAcb-Sp0
272	Neu5Aca2-6Galb1-4Glcb-Sp0
273	Neu5Aca2-6Galb1-4Glcb-Sp8
274	Neu5Aca2-6Galb-Sp8
275	Neu5Aca2-8Neu5Aca-Sp8
276	Neu5Aca2-8Neu5Aca2-3Galb1-4Glcb-Sp0
277	Galb1-3(Fuca1-4)GlcNAcb1-3Galb1-3(Fuca1-4)GlcNAcb-Sp0
278	Neu5Acb2-6GalNAca-Sp8
279	Neu5Acb2-6Galb1-4GlcNAcb-Sp8
280	Neu5Gca2-3Galb1-3(Fuca1-4)GlcNAcb-Sp0
281	Neu5Gca2-3Galb1-3GlcNAcb-Sp0
282	Neu5Gca2-3Galb1-4(Fuca1-3)GlcNAcb-Sp0
283	Neu5Gca2-3Galb1-4GlcNAcb-Sp0
284	Neu5Gca2-3Galb1-4Glcb-Sp0
285	Neu5Gca2-6GalNAca-Sp0
286	Neu5Gca2-6Galb1-4GlcNAcb-Sp0
287	Neu5Gca-Sp8
288	Neu5Aca2-3Galb1-4GlcNAcb1-6(Galb1-3)GalNAca-Sp14
289	Galb1-3GlcNAcb1-3Galb1-3GlcNAcb-Sp0
290	Galb1-4(Fuca1-3)(6S)GlcNAcb-Sp0
291	Galb1-4(Fuca1-3)(6S)Glcb-Sp0
292	Galb1-4(Fuca1-3)GlcNAcb1-3Galb1-3(Fuca1-4)GlcNAcb-Sp0
293	Galb1-4GlcNAcb1-3Galb1-3GlcNAcb-Sp0
294	Neu5Aca2-3Galb1-3GlcNAcb1-3Galb1-3GlcNAcb-Sp0
295	Neu5Aca2-3Galb1-4GlcNAcb1-3Galb1-3GlcNAcb-Sp0
296	4S(3S)Galb1-4GlcNAcb-Sp0
297	(6S)Galb1-4(6S)GlcNAcb-Sp0
298	(6P)Glcb-Sp10
299	Neu5Aca2-3Galb1-4(Fuca1-3)GlcNAcb1-6(Galb1-3)GalNAca-Sp14
300	Galb1-3Galb1-4GlcNAcb-Sp8
301	Neu5Aca2-6Galb1-4GlcNAcb1-2Mana1-6(Galb1-4GlcNAcb1-2Mana1-3)Manb1-4GlcNAcb1-4GlcNAcb-Sp12
302	Galb1-4GlcNAcb1-6(Galb1-4GlcNAcb1-3)Galb1-4GlcNAc-Sp0
303	GlcNAcb1-6(Galb1-4GlcNAcb1-3)Galb1-4GlcNAc-Sp0
304	Galb1-4GlcNAca1-6Galb1-4GlcNAcb-Sp0
305	Galb1-4GlcNAcb1-6Galb1-4GlcNAcb-Sp0
306	GalNAcb1-3Galb-Sp8
307	GlcAb1-3GlcNAcb-Sp8
308	Neu5Aca2-6Galb1-4GlcNAcb1-2Mana1-6(GlcNAcb1-2Mana1-3)Manb1-4GlcNAcb1-4GlcNAcb-Sp12
309	GlcNAcb1-3Man-Sp10
310	GlcNAcb1-4GlcNAcb-Sp10

Chart Number	Structure on Masterlist
311	GlcNAcb1-4GlcNAcb-Sp12
312	MurNAcb1-4GlcNAcb-Sp10
313	Mana1-6Manb-Sp10
314	Mana1-6(Mana1-3)Mana1-6(Mana1-3)Manb-Sp10
315	Mana1-2Mana1-6(Mana1-3)Mana1-6(Mana1-2Mana1-2Mana1-3)Mana-Sp9
316	Mana1-2Mana1-6(Mana1-2Mana1-3)Mana1-6(Mana1-2Mana1-2Mana1-3)Mana-Sp9
317	Neu5Aca2-3Galb1-4GlcNAcb1-6(Neu5Aca2-3Galb1-3)GalNAca-Sp14
318	Neu5Aca2-6Galb1-4GlcNAcb1-2Mana1-6(Neu5Aca2-3Galb1-4GlcNAcb1-2Mana1-3)Manb1-4GlcNAcb1-4GlcNAcb-Sp12
319	Galb1-4GlcNAcb1-2Mana1-6(Neu5Aca2-6Galb1-4GlcNAcb1-2Mana1-3)Manb1-4GlcNAcb1-4GlcNAcb-Sp12
320	GlcNAcb1-2Mana1-6(Neu5Aca2-6Galb1-4GlcNAcb1-2Mana1-3)Manb1-4GlcNAcb1-4GlcNAcb-Sp12
321	Neu5Aca2-8Neu5Acb-Sp17
322	Neu5Aca2-8Neu5Aca2-8Neu5Acb-Sp8
323	Neu5Gcb2-6Galb1-4GlcNAc-Sp8
324	Galb1-3GlcNAcb1-2Mana1-6(Galb1-3GlcNAcb1-2Mana1-3)Manb1-4GlcNAcb1-4GlcNAcb-Sp19
325	Neu5Aca2-3Galb1-4GlcNAcb1-2Mana1-6(Neu5Aca2-3Galb1-4GlcNAcb1-2Mana1-3)Manb1-4GlcNAcb1-4GlcNAcb-Sp12
326	Neu5Aca2-3Galb1-4GlcNAcb1-2Mana1-6(Neu5Aca2-6Galb1-4GlcNAcb1-2Mana1-3)Manb1-4GlcNAcb1-4GlcNAcb-Sp12
327	Galb1-4(Fuca1-3)GlcNAcb1-2Mana1-6(Galb1-4(Fuca1-3)GlcNAcb1-2Mana1-3)Manb1-4GlcNAcb1-4GlcNAcb-Sp20
328	Neu5,9Ac2a2-3Galb1-4GlcNAcb-Sp0
329	Neu5,9Ac2a2-3Galb1-3GlcNAcb-Sp0
330	Neu5Aca2-6Galb1-4GlcNAcb1-3Galb1-3GlcNAcb-Sp0
331	Neu5Aca2-3Galb1-3(Fuca1-4)GlcNAcb1-3Galb1-3(Fuca1-4)GlcNAcb-Sp0
332	Neu5Aca2-6Galb1-4GlcNAcb1-3Galb1-4GlcNAcb1-3Galb1-4GlcNAcb-Sp0
333	Gala1-4Galb1-4GlcNAcb1-3Galb1-4Glcb-Sp0
334	GalNAcb1-3Gala1-4Galb1-4GlcNAcb1-3Galb1-4Glcb-Sp0
335	GalNAca1-3(Fuca1-2)Galb1-4GlcNAcb1-3Galb1-4GlcNAcb-Sp0
336	GalNAca1-3(Fuca1-2)Galb1-4GlcNAcb1-3Galb1-4GlcNAcb1-3Galb1-4GlcNAcb-Sp0
337	Neu5Aca2-3Galb1-4(Fuca1-3)GlcNAcb1-6(Neu5Aca2-3Galb1-3)GalNAc-Sp14
338	GlcNAca1-4Galb1-4GlcNAcb1-3Galb1-4GlcNAcb1-3Galb1-4GlcNAcb-Sp0
339	GlcNAca1-4Galb1-4GlcNAcb-Sp0
340	GlcNAca1-4Galb1-3GlcNAcb-Sp0
341	GlcNAca1-4Galb1-4GlcNAcb1-3Galb1-4Glcb-Sp0
342	GlcNAca1-4Galb1-4GlcNAcb1-3Galb1-4(Fuca1-3)GlcNAcb1-3Galb1-4(Fuca1-3)GlcNAcb-Sp0
343	GlcNAca1-4Galb1-4GlcNAcb1-3Galb1-4GlcNAcb-Sp0
344	GlcNAca1-4Galb1-3GalNAc-Sp14
345	Neu5Aca2-6Galb1-4GlcNAcb1-2Mana1-6(Mana1-3)Manb1-4GlcNAcb1-4GlcNAc-Sp12
346	Mana1-6(Neu5Aca2-6Galb1-4GlcNAcb1-2Mana1-3)Manb1-4GlcNAcb1-4GlcNAc-Sp12
347	Neu5Aca2-6Galb1-4GlcNAcb1-2Mana1-6Manb1-4GlcNAcb1-4GlcNAc-Sp12
348	Neu5Aca2-6Galb1-4GlcNAcb1-2Mana1-3Manb1-4GlcNAcb1-4GlcNAc-Sp12
349	Galb1-4GlcNAcb1-2Mana1-3Manb1-4GlcNAcb1-4GlcNAc-Sp12
350	Galb1-4GlcNAcb1-2Mana1-6Manb1-4GlcNAcb1-4GlcNAc-Sp12
351	Mana1-6(Galb1-4GlcNAcb1-2Mana1-3)Manb1-4GlcNAcb1-4GlcNAcb-Sp12
352	GlcNAcb1-2Mana1-6(GlcNAcb1-2Mana1-3)Manb1-4GlcNAcb1-4(Fuca1-6)GlcNAcb-Sp22
353	Galb1-4GlcNAcb1-2Mana1-6(Galb1-4GlcNAcb1-2Mana1-3)Manb1-4GlcNAcb1-4(Fuca1-6)GlcNAcb-Sp22
354	Galb1-3GlcNAcb1-2Mana1-6(Galb1-3GlcNAcb1-2Mana1-3)Manb1-4GlcNAcb1-4(Fuca1-6)GlcNAcb-Sp22
355	(6S)GlcNAcb1-3Galb1-4GlcNAcb-Sp0
356	KDNa2-3Galb1-4(Fuca1-3)GlcNAc-Sp0
357	KDNa2-6Galb1-4GlcNAc-Sp0
358	KDNa2-3Galb1-4Glc-Sp0
359	KDNa2-3Galb1-3GalNAca-Sp14
360	Fuca1-2Galb1-3GlcNAcb1-2Mana1-6(Fuca1-2Galb1-3GlcNAcb1-2Mana1-3)Manb1-4GlcNAcb1-4GlcNAcb-Sp20
361	Fuca1-2Galb1-4GlcNAcb1-2Mana1-6(Fuca1-2Galb1-4GlcNAcb1-2Mana1-3)Manb1-4GlcNAcb1-4GlcNAcb-Sp20
362	Fuca1-2Galb1-4(Fuca1-3)GlcNAcb1-2Mana1-6(Fuca1-2Galb1-4(Fuca1-3)GlcNAcb1-2Mana1-3)Manb1-4GlcNAcb1-4GlcNAb-Sp20
363	Gala1-3Galb1-4GlcNAcb1-2Mana1-6(Gala1-3Galb1-4GlcNAcb1-2Mana1-3)Manb1-4GlcNAcb1-4GlcNAcb-Sp20
364	Galb1-4GlcNAcb1-2Mana1-6(Mana1-3)Manb1-4GlcNAcb1-4GlcNAcb-Sp12
365	Fuca1-4(Galb1-3)GlcNAcb1-2Mana1-6(Fuca1-4(Galb1-3)GlcNAcb1-2Mana1-3)Manb1-4GlcNAcb1-4(Fuca1-6)GlcNAcb-Sp22
366	Neu5Aca2-6GlcNAcb1-4GlcNAc-Sp21

Chart Number	Structure on Masterlist
367	Neu5Aca2-6GlcNAcb1-4GlcNAcb1-4GlcNAc-Sp21
368	Galb1-4(Fuca1-3)GlcNAcb1-6(Fuca1-2Galb1-4GlcNAcb1-3)Galb1-4Glc-Sp21
369	Galb1-4GlcNAcb1-2Mana1-6(Galb1-4GlcNAcb1-4(Galb1-4GlcNAcb1-2)Mana1-3)Manb1-4GlcNAcb1-4GlcNAc-Sp21
370	GalNAca1-3(Fuca1-2)Galb1-4GlcNAcb1-2Mana1-6(GalNAca1-3(Fuca1-2)Galb1-4GlcNAcb1-2Mana1-3)Manb1-4GlcNAcb1-4GlcNAcb-Sp20
371	Gala1-3(Fuca1-2)Galb1-4GlcNAcb1-2Mana1-6(Gala1-3(Fuca1-2)Galb1-4GlcNAcb1-2Mana1-3)Manb1-4GlcNAcb1-4GlcNAcb-Sp20
372	Gala1-3Galb1-4(Fuca1-3)GlcNAcb1-2Mana1-6(Gala1-3Galb1-4(Fuca1-3)GlcNAcb1-2Mana1-3)Manb1-4GlcNAcb1-4GlcNAcb-Sp20
373	GalNAca1-3(Fuca1-2)Galb1-3GlcNAcb1-2Mana1-6(GalNAca1-3(Fuca1-2)Galb1-3GlcNAcb1-2Mana1-3)Manb1-4GlcNAcb1-4GlcNAcb-Sp20
374	Gala1-3(Fuca1-2)Galb1-3GlcNAcb1-2Mana1-6(Gala1-3(Fuca1-2)Galb1-3GlcNAcb1-2Mana1-3)Manb1-4GlcNAcb1-4GlcNAcb-Sp20
375	Fuca1-4(Fuca1-2Galb1-3)GlcNAcb1-2Mana1-3(Fuca1-4(Fuca1-2Galb1-3)GlcNAcb1-2Mana1-3)Manb1-4GlcNAcb1-4GlcNAcb-Sp19
376	Neu5Aca2-3Galb1-4GlcNAcb1-3GalNAc-Sp14
377	Neu5Aca2-6Galb1-4GlcNAcb1-3GalNAc-Sp14
378	Neu5Aca2-3Galb1-4(Fuca1-3)GlcNAcb1-3GalNAca-Sp14
379	GalNAcb1-4GlcNAcb1-2Mana1-6(GalNAcb1-4GlcNAcb1-2Mana1-3)Manb1-4GlcNAcb1-4GlcNAc-Sp12
380	Galb1-3GalNAca1-3(Fuca1-2)Galb1-4Glc-Sp0
381	Galb1-3GalNAca1-3(Fuca1-2)Galb1-4GlcNAc-Sp0
382	Galb1-3GlcNAcb1-3Galb1-4GlcNAcb1-6(Galb1-3GlcNAcb1-3)Galb1-4Glcb-Sp0
383	Galb1-4(Fuca1-3)GlcNAcb1-6(Galb1-3GlcNAcb1-3)Galb1-4Glc-Sp21
384	Galb1-4GlcNAcb1-6(Fuca1-4(Fuca1-2Galb1-3)GlcNAcb1-3)Galb1-4Glc-Sp21
385	Galb1-4(Fuca1-3)GlcNAcb1-6(Fuca1-4(Fuca1-2Galb1-3)GlcNAcb1-3)Galb1-4Glc-Sp21
386	Galb1-3GlcNAcb1-3Galb1-4(Fuca1-3)GlcNAcb1-6(Galb1-3GlcNAcb1-3)Galb1-4Glc-Sp21
387	Galb1-4GlcNAcb1-6(Galb1-4GlcNAcb1-2)Mana1-6(Galb1-4GlcNAcb1-4(Galb1-4GlcNAcb1-2)Mana1-3)Manb1-4GlcNAcb1-4GlcNAcb-Sp21
388	GlcNAcb1-2Mana1-6(GlcNAcb1-4(GlcNAcb1-2)Mana1-3)Manb1-4GlcNAcb1-4GlcNAc-Sp21
389	Fuca1-2Galb1-3GalNAca1-3(Fuca1-2)Galb1-4Glcb-Sp0
390	Fuca1-2Galb1-3GalNAca1-3(Fuca1-2)Galb1-4GlcNAcb-Sp0
391	Galb1-3GlcNAcb1-3GalNAca-Sp14
392	GalNAcb1-4(Neu5Aca2-3)Galb1-4GlcNAcb1-3GalNAca-Sp14
393	GalNAca1-3(Fuca1-2)Galb1-3GalNAca1-3(Fuca1-2)Galb1-4GlcNAcb-Sp0
394	Gala1-3Galb1-3GlcNAcb1-2Mana1-6(Gala1-3Galb1-3GlcNAcb1-2Mana1-3)Manb1-4GlcNAcb1-4GlcNAc-Sp19
395	Gala1-3Galb1-3(Fuca1-4)GlcNAcb1-2Mana1-6(Gala1-3Galb1-3(Fuca1-4)GlcNAcb1-2Mana1-3)Manb1-4GlcNAcb1-4GlcNAc-Sp19
396	Neu5Aca2-3Galb1-3GlcNAcb1-2Mana1-6(Neu5Aca2-3Galb1-3GlcNAcb1-2Mana1-3)Manb1-4GlcNAcb1-4GlcNAc-Sp19
397	GlcNAcb1-2Mana1-6(Galb1-4GlcNAcb1-2Mana1-3)Manb1-4GlcNAcb1-4GlcNAc-Sp12
398	Galb1-4GlcNAcb1-2Mana1-6(GlcNAcb1-2Mana1-3)Manb1-4GlcNAcb1-4GlcNAc-Sp12
399	Neu5Aca2-3Galb1-3GlcNAcb1-3GalNAca-Sp14
400	Fuca1-2Galb1-4GlcNAcb1-3GalNAca-Sp14
401	Galb1-4(Fuca1-3)GlcNAcb1-3GalNAca-Sp14
402	GalNAca1-3GalNAcb1-3Gala1-4Galb1-4GlcNAcb-Sp0
403	Gala1-4Galb1-3GlcNAcb1-2Mana1-6(Gala1-4Galb1-3GlcNAcb1-2Mana1-3)Manb1-4GlcNAcb1-4GlcNAcb-Sp19
404	Gala1-4Galb1-4GlcNAcb1-2Mana1-6(Gala1-4Galb1-4GlcNAcb1-2Mana1-3)Manb1-4GlcNAcb1-4GlcNAcb-Sp24
405	Gala1-3Galb1-4GlcNAcb1-3GalNAca-Sp14
406	Galb1-3GlcNAcb1-6Galb1-4GlcNAcb-Sp0
407	Galb1-3GlcNAca1-6Galb1-4GlcNAcb-Sp0
408	GalNAcb1-3Gala1-6Galb1-4Glcb-Sp8
409	Gala1-3(Fuca1-2)Galb1-4(Fuca1-3)Glcb-Sp21
410	Galb1-4GlcNAcb1-6(Neu5Aca2-6Galb1-3GlcNAcb1-3)Galb1-4Glc-Sp21
411	Galb1-3GalNAcb1-4(Neu5Aca2-8Neu5Aca2-3)Galb1-4Glcb-Sp0
412	Neu5Aca2-3Galb1-3GalNAcb1-4(Neu5Aca2-8Neu5Aca2-3)Galb1-4Glcb-Sp0
413	Gala1-3(Fuca1-2)Galb1-4GlcNAcb1-3GalNAca-Sp14
414	GalNAca1-3(Fuca1-2)Galb1-4GlcNAcb1-3GalNAca-Sp14
415	GalNAca1-3GalNAcb1-3Gala1-4Galb1-4Glcb-Sp0
416	Fuca1-2Galb1-4(Fuca1-3)GlcNAcb1-3GalNAca-Sp14
417	Gala1-3(Fuca1-2)Galb1-4(Fuca1-3)GlcNAcb1-3GalNAc-Sp14
418	GalNAca1-3(Fuca1-2)Galb1-4(Fuca1-3)GlcNAcb1-3GalNAc-Sp14

Chart Number	Structure on Masterlist
419	Galb1-4(Fuca1-3)GlcNAcb1-2Mana1-6(Galb1-4(Fuca1-3)GlcNAcb1-2Mana1-3)Manb1-4GlcNAcb1-4(Fuca1-6)GlcNAcb-Sp22
420	Fuca1-2Galb1-4GlcNAcb1-2Mana1-6(Fuca1-2Galb1-4GlcNAcb1-2Mana1-3)Manb1-4GlcNAcb1-4(Fuca1-6)GlcNAcb-Sp22
421	GlcNAcb1-2(GlcNAcb1-6)Mana1-6(GlcNAcb1-2Mana1-3)Manb1-4GlcNAcb1-4GlcNAcb-Sp19
422	Fuca1-2Galb1-3GlcNAcb1-3GalNAc-Sp14
423	Gala1-3(Fuca1-2)Galb1-3GlcNAcb1-3GalNAc-Sp14
424	GalNAca1-3(Fuca1-2)Galb1-3GlcNAcb1-3GalNAc-Sp14
425	Gala1-3Galb1-3GlcNAcb1-3GalNAc-Sp14
426	Fuca1-2Galb1-3GlcNAcb1-2Mana1-6(Fuca1-2Galb1-3GlcNAcb1-2Mana1-3)Manb1-4GlcNAcb1-4(Fuca1-6)GlcNAcb-Sp22
427	Gala1-3(Fuca1-2)Galb1-4GlcNAcb1-2Mana1-6(Gala1-3(Fuca1-2)Galb1-4GlcNAcb1-2Mana1-3)Manb1-4GlcNAcb1-4(Fuca1-6)GlcNAcb-Sp22
428	Galb1-3GlcNAcb1-6(Galb1-3GlcNAcb1-2)Mana1-6(Galb1-3GlcNAcb1-2Mana1-3)Manb1-4GlcNAcb1-4GlcNAcb-Sp19
429	Galb1-4GlcNAcb1-6(Fuca1-2Galb1-3GlcNAcb1-3)Galb1-4Glc-Sp21
430	Fuca1-3GlcNAcb1-6(Galb1-4GlcNAcb1-3)Galb1-4Glc-Sp21
431	GlcNAcb1-2Mana1-6(GlcNAcb1-4)(GlcNAcb1-2Mana1-3)Manb1-4GlcNAcb1-4GlcNAc-Sp21
432	GlcNAcb1-2Mana1-6(GlcNAcb1-4)(GlcNAcb1-4(GlcNAcb1-2)Mana1-3)Manb1-4GlcNAcb1-4GlcNAc-Sp21
433	GlcNAcb1-6(GlcNAcb1-2)Mana1-6(GlcNAcb1-4)(GlcNAcb1-2Mana1-3)Manb1-4GlcNAcb1-4GlcNAc-Sp21
434	GlcNAcb1-6(GlcNAcb1-2)Mana1-6(GlcNAcb1-4)(GlcNAcb1-4(GlcNAcb1-2)Mana1-3)Manb1-4GlcNAcb1-4GlcNAc-Sp21
435	Galb1-4GlcNAcb1-2Mana1-6(GlcNAcb1-4)(Galb1-4GlcNAcb1-2Mana1-3)Manb1-4GlcNAcb1-4GlcNAc-Sp21
436	Galb1-4GlcNAcb1-2Mana1-6(GlcNAcb1-4)(Galb1-4GlcNAcb1-4(Galb1-4GlcNAcb1-2)Mana1-3)Manb1-4GlcNAcb1-4GlcNAc-Sp21
437	Galb1-4GlcNAcb1-6(Galb1-4GlcNAcb1-2)Mana1-6(GlcNAcb1-4)(Galb1-4GlcNAcb1-2Mana1-3)Manb1-4GlcNAcb1-4GlcNAc-Sp21
438	Galb1-4GlcNAcb1-6(Galb1-4GlcNAcb1-2)Mana1-6(GlcNAcb1-4)(Galb1-4GlcNAcb1-4(Galb1-4GlcNAcb1-2)Mana1-3)Manb1-4GlcNAcb1-4GlcNAc-Sp21
439	Galb1-4Galb-Sp10
440	Galb1-6Galb-Sp10
441	Neu5Aca2-3Galb1-4GlcNAcb1-3Galb-Sp8
442	GalNAcb1-6GalNAcb-Sp8
443	(6S)Galb1-3GlcNAcb-Sp0
444	(6S)Galb1-3(6S)GlcNAc-Sp0
445	Fuca1-2Galb1-4 GlcNAcb1-2Mana1-6(Fuca1-2Galb1-4GlcNAcb1-2(Fuca1-2Galb1-4GlcNAcb1-4)Mana1-3)Manb1-4GlcNAcb1-4GlcNAcb-Sp12
446	Fuca1-2Galb1-4(Fuca1-3)GlcNAcb1-2Mana1-6(Fuca1-2Galb1-4(Fuca1-3)GlcNAcb1-4(Fuca1-2Galb1-4(Fuca1-3)GlcNAcb1-2)Mana1-3)Manb1-4GlcNAcb1-4GlcNAcb-Sp12
447	Galb1-4(Fuca1-3)GlcNAcb1-6GalNAc-Sp14
448	Galb1-4GlcNAcb1-2Mana-Sp0
449	Fuca1-2Galb1-4GlcNAcb1-6(Fuca1-2Galb1-4GlcNAcb1-3)GalNAc-Sp14
450	Gala1-3Fuca1-2Galb1-4GlcNAcb1-6(Gala1-3Fuca1-2Galb1-4GlcNAcb1-3)GalNAc-Sp14
451	GalNAca1-3(Fuca1-2)Galb1-4GlcNAcb1-6(GalNAca1-3(Fuca1-2)Galb1-4GlcNAcb1-3)GalNAc-Sp14
452	Neu5Aca2-8Neu5Aca2-3Galb1-3GalNAcb1-4(Neu5Aca2-8Neu5Aca2-3)Galb1-4Glcb-Sp0
453	GalNAcb1-4Galb1-4Glcb-Sp0
454	GalNAca1-3(Fuca1-2)Galb1-4GlcNAcb1-2Mana1-6(GalNAca1-3(Fuca1-2)Galb1-4GlcNAcb1-2Mana1-3)Manb1-4GlcNAcb1-4(Fuca1-6)GlcNAcb-Sp22
455	Gala1-3(Fuca1-2)Galb1-3GlcNAcb1-2Mana1-6(Gala1-3(Fuca1-2)Galb1-3GlcNAcb1-2Mana1-3)Manb1-4GlcNAcb1-4(Fuca1-6)GlcNAcb-Sp22
456	Neu5Aca2-6Galb1-4GlcNAcb1-6(Fuca1-2Galb1-3GlcNAcb1-3)Galb1-4Glc-Sp21
457	GalNAca1-3(Fuca1-2)Galb1-3GlcNAcb1-2Mana1-6(GalNAca1-3(Fuca1-2)Galb1-3GlcNAcb1-2Mana1-3)Manb1-4GlcNAcb1-4(Fuca1-6)GlcNAcb-Sp22
458	Galb1-4GlcNAcb1-6(Galb1-4GlcNAcb1-2)Mana1-6(Galb1-4GlcNAcb1-2Mana1-3)Manb1-4GlcNAcb1-4GlcNAcb-Sp19
459	Neu5Aca2-3Galb1-4GlcNAcb1-2Mana1-6(GlcNAcb1-4)(Neu5Aca2-3Galb1-4GlcNAcb1-2Mana1-3)Manb1-4GlcNAcb1-4GlcNAcb-Sp21
460	Neu5Aca2-3Galb1-4GlcNAcb1-4Mana1-6(GlcNAcb1-4)(Neu5Aca2-3Galb1-4GlcNAcb1-4(Neu5Aca2-3Galb1-4GlcNAcb1-2)Mana1-3)Manb1-4GlcNAcb1-4GlcNAcb-Sp21
461	Neu5Aca2-3Galb1-4GlcNAcb1-6(Neu5Aca2-3Galb1-4GlcNAcb1-2)Mana1-6(GlcNAcb1-4)(Neu5Aca2-3Galb1-4GlcNAcb1-2Mana1-3)Manb1-4GlcNAcb1-4GlcNAcb-Sp21

Chart Number	Structure on Masterlist
462	Neu5Aca2-3Galb1-4GlcNAcb1-6(Neu5Aca2-3Galb1-4GlcNAcb1-2)Mana1-6(GlcNAcb1-4)(Neu5Aca2-3Galb1-4GlcNAcb1-4(Neu5Aca2-3Galb1-4GlcNAcb1-2)Mana1-3)Manb1-4GlcNAcb1-4GlcNAcb-Sp21
463	Neu5Aca2-6Galb1-4GlcNAcb1-2Mana1-6(GlcNAcb1-4)(Neu5Aca2-6Galb1-4GlcNAcb1-2Mana1-3)Manb1-4GlcNAcb1-4GlcNAcb-Sp21
464	Neu5Aca2-6Galb1-4GlcNAcb1-4Mana1-6(GlcNAcb1-4)(Neu5Aca2-6Galb1-4GlcNAcb1-4(Neu5Aca2-6Galb1-4GlcNAcb1-2)Mana1-3)Manb1-4GlcNAcb1-4GlcNAcb-Sp21
465	Neu5Aca2-6Galb1-4GlcNAcb1-6(Neu5Aca2-6Galb1-4GlcNAcb1-2)Mana1-6(GlcNAcb1-4)(Neu5Aca2-6Galb1-4GlcNAcb1-2Mana1-3)Manb1-4GlcNAcb1-4GlcNAcb-Sp21
466	Neu5Aca2-6Galb1-4GlcNAcb1-6(Neu5Aca2-6Galb1-4GlcNAcb1-2)Mana1-6(GlcNAcb1-4)(Neu5Aca2-6Galb1-4GlcNAcb1-4(Neu5Aca2-6Galb1-4GlcNAcb1-2)Mana1-3)Manb1-4GlcNAcb1-4GlcNAcb-Sp21
467	Gala1-3(Fuca1-2)Galb1-3GalNAca-Sp8
468	Gala1-3(Fuca1-2)Galb1-3GalNAcb-Sp8
469	Glca1-6Glca1-6Glca1-6Glcb-Sp10
470	Glca1-4Glca1-4Glca1-4Glcb-Sp10
471	Neu5Aca2-3Galb1-4GlcNAcb1-6(Neu5Aca2-3Galb1-4GlcNAcb1-3)GalNAca-Sp14
472	Fuca1-2Galb1-4(Fuca1-3)GlcNAcb1-2Mana1-6(Fuca1-2Galb1-4(Fuca1-3)GlcNAcb1-2Mana1-3)Manb1-4GlcNAcb1-4(Fuca1-6)GlcNAcb-Sp24
473	Fuca1-2Galb1-3(Fuca1-4)GlcNAcb1-2Mana1-6(Fuca1-2Galb1-3(Fuca1-4)GlcNAcb1-2Mana1-3)Manb1-4GlcNAcb1-4(Fuca1-6)GlcNAcb1-4(Fuca1-6)GlcNAcb-Sp19
474	Neu5Aca2-3Galb1-3GlcNAcb1-6(Neu5Aca2-3Galb1-4GlcNAcb1-2)Mana1-6(Neu5Aca2-3Galb1-3GlcNAcb1-2Mana1-3)Manb1-4GlcNAcb1-4GlcNAcb-Sp19
475	GlcNAcb1-6(GlcNAcb1-2)Mana1-6(GlcNAcb1-2Mana1-3)Manb1-4GlcNAcb1-4(Fuca1-6)GlcNAcb-Sp24
476	Galb1-3GlcNAcb1-2Mana1-6(GlcNAcb1-4)(Galb1-3GlcNAcb1-2Mana1-3)Manb1-4GlcNAcb1-4GlcNAcb-Sp21
477	Neu5Aca2-6Galb1-4GlcNAcb1-6(Galb1-3GlcNAcb1-3)Galb1-4Glcb-Sp21
478	Neu5Aca2-3Galb1-4GlcNAcb1-2Mana-Sp0
479	Neu5Aca2-3Galb1-4GlcNAcb1-6GalNAca-Sp14
480	Neu5Aca2-6Galb1-4GlcNAcb1-6GalNAca-Sp14
481	Neu5Aca2-6Galb1-4 GlcNAcb1-6(Neu5Aca2-6Galb1-4GlcNAcb1-3)GalNAca-Sp14
482	Neu5Aca2-6Galb1-4GlcNAcb1-2Mana1-6(Neu5Aca2-6Galb1-4GlcNAcb1-2Mana1-3)Manb1-4GlcNAcb1-4(Fuca1-6)GlcNAcb-Sp24
483	Neu5Aca2-3Galb1-4GlcNAcb1-2Mana1-6(Neu5Aca2-3Galb1-4GlcNAcb1-2Mana1-3)Manb1-4GlcNAcb1-4(Fuca1-6)GlcNAcb-Sp24
484	Mana1-6(Mana1-3)Manb1-4GlcNAcb1-4(Fuca1-6)GlcNAcb-Sp19
485	Galb1-4GlcNAcb1-6(Galb1-4GlcNAcb1-2)Mana1-6(Galb1-4GlcNAcb1-2Mana1-3)Manb1-4GlcNAcb1-4(Fuca1-6)GlcNAcb-Sp24
486	Neu5Aca2-3Galb1-3GlcNAcb1-2Mana1-6(GlcNAcb1-4)(Neu5Aca2-3Galb1-3GlcNAcb1-2Mana1-3)Manb1-4GlcNAcb1-4GlcNAc-Sp21
487	Neu5Aca2-6Galb1-4GlcNAcb1-6(Fuca1-2Galb1-4(Fuca1-3)GlcNAcb1-3)Galb1-4Glc-Sp21
488	Galb1-3GlcNAcb1-6GalNAca-Sp14
489	Gala1-3Galb1-3GlcNAcb1-6GalNAca-Sp14
490	Galb1-3(Fuca1-4)GlcNAcb1-6GalNAca-Sp14
491	Neu5Aca2-3Galb1-3GlcNAcb1-6GalNAca-Sp14
492	(3S)Galb1-3(Fuca1-4)GlcNAcb-Sp0
493	Galb1-4(Fuca1-3)GlcNAcb1-6(Neu5Aca2-6(Neu5Aca2-3Galb1-3)GlcNAcb1-3)Galb1-4Glc-Sp21
494	Fuca1-2Galb1-4GlcNAcb1-6GalNAca-Sp14
495	Gala1-3Galb1-4GlcNAcb1-6GalNAca-Sp14
496	Galb1-4(Fuca1-3)GlcNAcb1-2Mana-Sp0
497	Fuca1-2(6S)Galb1-3GlcNAcb-Sp0
498	Gala1-3(Fuca1-2)Galb1-4GlcNAcb1-6GalNAca-Sp14
499	Fuca1-2Galb1-4GlcNAcb1-2Mana-Sp0
500	Fuca1-2Galb1-3(6S)GlcNAcb-Sp0
501	Fuca1-2(6S)Galb1-3(6S)GlcNAcb-Sp0
502	Neu5Aca2-6GalNAcb1-4(6S)GlcNAcb-Sp8
503	GalNAcb1-4(Fuca1-3)(6S)GlcNAcb-Sp8
504	(3S)GalNAcb1-4(Fuca1-3)GlcNAcb-Sp8
505	Fuca1-2Galb1-3GlcNAcb1-6(Fuca1-2Galb1-3GlcNAcb1-3)GalNAca-Sp14
506	GalNAca1-3(Fuca1-2)Galb1-3GlcNAcb1-6GalNAca-Sp14
507	GlcNAcb1-6(GlcNAcb1-2)Mana1-6(GlcNAcb1-4)(GlcNAcb1-4(GlcNAcb1-2)Mana1-3)Manb1-4GlcNAcb1-4(Fuca1-6)GlcNAc-Sp21
508	Galb1-4GlcNAcb1-6(Galb1-4GlcNAcb1-2)Mana1-6(GlcNAcb1-4)Galb1-4GlcNAcb1-4(Gal b1-4GlcNAcb1-2)Mana1-3)Manb1-4GlcNAcb1-4(Fuca1-6)GlcNAc-Sp21
509	Galb1-3GlcNAca1-3Galb1-4GlcNAcb-Sp8

Chart Number	Structure on Masterlist
510	Galb1-3(6S)GlcNAcb-Sp8
511	(6S)(4S)GalNAcb1-4GlcNAc-Sp8
512	(6S)GalNAcb1-4GlcNAc-Sp8
513	(3S)GalNAcb1-4(3S)GlcNAc-Sp8
514	GalNAcb1-4(6S)GlcNAc-Sp8
515	(3S)GalNAcb1-4GlcNAc-Sp8
516	(4S)GalNAcb-Sp10
517	Galb1-4(6P)GlcNAcb-Sp0
518	(6P)Galb1-4GlcNAcb-SP0
519	GalNAca1-3(Fuca1-2)Galb1-4GlcNAcb1-6GalNAc-Sp14
520	Neu5Aca2-6Galb1-4GlcNAcb1-2Man-Sp0
521	Gala1-3Galb1-4GlcNAcb1-2Mana-Sp0
522	Gala1-3(Fuca1-2)Galb1-4GlcNAcb1-2Mana-Sp0
523	GalNAca1-3(Fuca1-2)Galb1-4 GlcNAcb1-2Mana-Sp0
524	Galb1-3GlcNAcb1-2Mana-Sp0
525	Gala1-3(Fuca1-2)Galb1-3GlcNAcb1-6GalNAc-Sp14
526	Neu5Aca2-3Galb1-3GlcNAcb1-2Mana-Sp0
527	Gala1-3Galb1-3GlcNAcb1-2Mana-Sp0
528	GalNAcb1-4GlcNAcb1-2Mana-Sp0
529	Neu5Aca2-3Galb1-3GlcNAcb1-4Galb1-4Glcb-Sp0
530	GlcNAcb1-2 Mana1-6(GlcNAcb1-4)(GlcNAcb1-2Mana1-3)Manb1-4GlcNAcb1-4(Fuca1-6)GlcNAc-Sp21
531	Galb1-4GlcNAcb1-2 Mana1-6(GlcNAcb1-4)(Galb1-4GlcNAcb1-2Mana1-3)Manb1-4GlcNAcb1-4(Fuca1-6)GlcNAc-Sp21
532	Galb1-4GlcNAcb1-2 Mana1-6(Galb1-4GlcNAcb1-4)(Galb1-4GlcNAcb1-2Mana1-3)Manb1-4GlcNAcb1-4(Fuca1-6)GlcNAc-Sp21
533	Fuca1-4(Galb1-3)GlcNAcb1-2 Mana-Sp0
534	Neu5Aca2-3Galb1-4(Fuca1-3)GlcNAcb1-2Mana-Sp0
535	GlcNAcb1-3Galb1-4GlcNAcb1-6(GlcNAcb1-3)Galb1-4GlcNAc-Sp0
536	GalNAca1-3(Fuca1-2)Galb1-3GalNAcb1-3Gala1-4Galb1-4Glc-Sp21
537	Gala1-3(Fuca1-2)Galb1-3GalNAcb1-3Gala1-4Galb1-4Glc-Sp21
538	Galb1-3GalNAcb1-3Gal-Sp21
539	GlcNAcb1-3Galb1-4GlcNAcb1-2Mana1-6(GlcNAcb1-3Galb1-4GlcNAcb1-2Mana1-3)Manb1-4GlcNAcb1-4GlcNAcb-Sp12
540	GlcNAcb1-3Galb1-4GlcNAcb1-2Mana1-6(GlcNAcb1-3Galb1-4GlcNAcb1-2Mana1-3)Manb1-4GlcNAcb1-4GlcNAcb-Sp25
541	Galb1-4GlcNAcb1-3Galb1-4GlcNAcb1-2Mana1-6(Galb1-4GlcNAcb1-3Galb1-4GlcNAcb1-2Mana1-3)Manb1-4GlcNAcb1-4GlcNAcb-Sp12
542	Galb1-4GlcNAcb1-3Galb1-4GlcNAcb1-2Mana1-6(Galb1-4GlcNAcb1-3Galb1-4GlcNAcb1-2Mana1-3)Manb1-4GlcNAcb1-4GlcNAcb-Sp24
543	Neu5Gca2-3Galb1-4GlcNAcb1-3Galb1-4GlcNAcb1-2Mana1-6(Neu5Gca2-3Galb1-4GlcNAcb1-3Galb1-4GlcNAcb1-2Mana1-3)Manb1-4GlcNAcb1-4GlcNAcb-Sp24
544	Fuca1-2Galb1-4GlcNAcb1-3Galb1-4GlcNAcb1-2Mana1-6(Fuca1-2Galb1-4GlcNAcb1-3Galb1-4GlcNAcb1-2Mana1-3)Manb1-4GlcNAcb1-4GlcNAcb-Sp24
545	GlcNAcb1-3Galb1-4GlcNAcb1-3Galb1-4GlcNAcb1-2Mana1-6(GlcNAcb1-3Galb1-4GlcNAcb1-3Galb1-4GlcNAcb1-2Mana1-3)Manb1-4GlcNAcb1-4GlcNAcb-Sp12
546	GlcNAcb1-3Galb1-4GlcNAcb1-3Galb1-4GlcNAcb1-2Mana1-6(GlcNAcb1-3Galb1-4GlcNAcb1-3Galb1-4GlcNAcb1-2Mana1-3)Manb1-4GlcNAcb1-4GlcNAcb-Sp25
547	Galb1-4GlcNAcb1-3Galb1-4GlcNAcb1-3Galb1-4GlcNAcb1-2Mana1-6(Galb1-4GlcNAcb1-3Galb1-4GlcNAcb1-3Galb1-4GlcNAcb1-2Mana1-3)Manb1-4GlcNAcb1-4GlcNAcb-Sp12
548	Galb1-4GlcNAcb1-3Galb1-4GlcNAcb1-3Galb1-4GlcNAcb1-2Mana1-6(Galb1-4GlcNAcb1-3Galb1-4GlcNAcb1-3Galb1-4GlcNAcb1-2Mana1-3)Manb1-4GlcNAcb1-4GlcNAcb-Sp24
549	GlcNAcb1-3Galb1-4GlcNAcb1-3Galb1-4GlcNAcb1-3Galb1-4GlcNAcb1-2Mana1-6(GlcNAcb1-3Galb1-4GlcNAcb1-3Galb1-4GlcNAcb1-3Galb1-4GlcNAcb1-2Mana1-3)Manb1-4GlcNAcb1-4GlcNAcb-Sp25
550	Galb1-4GlcNAcb1-3Galb1-4GlcNAcb1-3Galb1-4GlcNAcb1-3Galb1-4GlcNAcb1-2Mana1-6(Galb1-4GlcNAcb1-3Galb1-4GlcNAcb1-3Galb1-4GlcNAcb1-3Galb1-4GlcNAcb1-2Mana1-3)Manb1-4GlcNAcb1-4GlcNAcb-Sp25
551	Galb1-3GlcNAcb1-3Galb1-4GlcNAcb1-2Mana1-6(Galb1-3GlcNAcb1-3Galb1-4GlcNAcb1-2Mana1-3)Manb1-4GlcNAcb1-4GlcNAc-Sp25
552	Neu5Gca2-8Neu5Gca2-3Galb1-4GlcNAc-Sp0
553	Neu5Aca2-8Neu5Gca2-3Galb1-4GlcNAc-Sp0
554	Neu5Gca2-8Neu5Aca2-3Galb1-4GlcNAc-Sp0
555	Neu5Gca2-8Neu5Gca2-3Galb1-4GlcNAcb1-3Galb1-4GlcNAc-Sp0
556	Neu5Gca2-8Neu5Gca2-6Galb1-4GlcNAc-Sp0

Chart Number	Structure on Masterlist
557	Neu5Aca2-8Neu5Aca2-3Galb1-4GlcNAc-Sp0
558	GlcNAcb1-3Galb1-4GlcNAcb1-6(GlcNAcb1-3Galb1-4GlcNAcb1-2)Mana1-6(GlcNAcb1-3Galb1-4GlcNAcb1-2Man a1-3)Manb1-4GlcNAcb1-4GlcNAc-Sp24
559	Galb1-4GlcNAcb1-3Galb1-4GlcNAcb1-6(Galb1-4GlcNAcb1-3Galb1-4GlcNAcb1-2)Mana1-6(Galb1-4GlcNAcb1-3Galb1-4GlcNAcb1-2Mana1-3)Mana1-4GlcNAcb1-4GlcNAc-Sp24
560	Gala1-3Galb1-4GlcNAcb1-2Mana1-6(Gala1-3Galb1-4GlcNAcb1-2Mana1-3)Manb1-4GlcNAcb1-4GlcNAc-Sp24
561	GlcNAcb1-3Galb1-4GlcNAcb1-6(GlcNAcb1-3Galb1-3)GalNAca-Sp14
562	GalNAcb1-3GlcNAcb-Sp0
563	GalNAcb1-4GlcNAcb1-3GalNAcb1-4GlcNAcb-Sp0
564	GlcNAcb1-3Galb1-4GlcNAcb1-3Galb1-4GlcNAcb1-3Galb1-4GlcNAcb1-3Galb1-4GlcNAcb1-2Mana1-6(GlcNAcb1-3Galb1-4GlcNAcb1-3Galb1-4GlcNAcb1-3Galb1-4GlcNAcb1-3Galb1-4GlcNAcb1-2Mana1-3)Manb1-4GlcNAcb1-4GlcNAcb-Sp25
565	Galb1-4GlcNAcb1-3Galb1-4GlcNAcb1-3Galb1-4GlcNAcb1-3Galb1-4GlcNAcb1-3Galb1-4GlcNAcb1-2Mana1-6(Galb1-4GlcNAcb1-3Galb1-4GlcNAcb1-3Galb1-4GlcNAcb1-3Galb1-4GlcNAcb1-3Galb1-4GlcNAcb1-2Mana1-3)Manb1-4GlcNAcb1-4GlcNAcb-Sp25
566	GlcNAb1-3Galb1-3GalNAc-Sp14
567	Galb1-3GlcNAcb1-6(Galb1-3)GalNAc-Sp14
568	Galb1-4GlcNAcb1-3Galb1-4GlcNAcb1-3Galb1-4GlcNAcb1-3Galb1-4GlcNAcb1-3Galb1-4GlcNAcb1-3Galb1-4GlcNAcb1-2Mana1-6(Galb1-4GlcNAcb1-3Galb1-4GlcNAcb1-3Galb1-4GlcNAcb1-3Galb1-4GlcNAcb1-3Galb1-4GlcNAcb1-3Galb1-4GlcNAcb1-2Mana1-3)Manb1-4GlcNAcb1-4GlcNAcb-Sp25
569	(3S)GlcAb1-3Galb1-4GlcNAcb1-3Galb1-4Glc-Sp0
570	(3S)GlcAb1-3Galb1-4GlcNAcb1-2Mana-Sp0
571	Galb1-3GlcNAcb1-3Galb1-4GlcNAcb1-3Galb1-4GlcNAcb1-6(Galb1-3GlcNAcb1-3Galb1-4GlcNAcb1-3Galb1-4GlcNAb1-2)Mana1-6(Galb1-3GlcNAcb1-3Galb1-4GlcNAcb1-3Galb1-4GlcNAcb1-2Mana1-3)Manb1-4GlcNAcb1-4(Fuca1-6)GlcNAcb-Sp24
572	Galb1-3GlcNAcb1-3Galb1-4GlcNAcb1-6(Galb1-3GlcNAcb1-3Galb1-4GlcNAb1-2)Mana1-6(Galb1-3GlcNAcb1-3Galb1-4GlcNAcb1-2Mana1-3)Manb1-4GlcNAcb1-4(Fuca1-6)GlcNAcb-Sp24
573	Neu5Aca2-8Neu5Aca2-3Galb1-3GalNAcb1-4(Neu5Aca2-3)Galb1-4Glc-Sp21
574	GlcNAcb1-3Galb1-4GlcNAcb1-2Mana1-6(GlcNAcb1-3Galb1-4GlcNAcb1-2Mana1-3)Manb1-4GlcNAcb1-4(Fuca1-6)GlcNAcb-Sp24
575	Galb1-4GlcNAcb1-3Galb1-4GlcNAcb1-2Mana1-6(Galb1-4GlcNAcb1-3Galb1-4GlcNAcb1-2Mana1-3)Manb1-4GlcNAcb1-4(Fuca1-6)GlcNAcb-Sp24
576	GlcNAcb1-3Galb1-4GlcNAcb1-3Galb1-4GlcNAcb1-2Mana1-6(GlcNAcb1-3Galb1-4GlcNAcb1-3Galb1-4GlcNAcb1-2Mana1-3)Manb1-4GlcNAcb1-4(Fuca1-6)GlcNAcb-Sp24
577	Galb1-4GlcNAcb1-3Galb1-4GlcNAcb1-3Galb1-4GlcNAcb1-2Mana1-6(Galb1-4GlcNAcb1-3Galb1-4GlcNAcb1-3Galb1-4GlcNAcb1-2Mana1-3)Manb1-4GlcNAcb1-4(Fuca1-6)GlcNAcb-Sp24
578	GlcNAcb1-3Galb1-4GlcNAcb1-3Galb1-4GlcNAcb1-3Galb1-4GlcNAcb1-2Mana1-6(GlcNAcb1-3Galb1-4GlcNAcb1-3Galb1-4GlcNAcb1-3Galb1-4GlcNAcb1-2Mana1-3)Manb1-4GlcNAcb1-4(Fuca1-6)GlcNAcb-Sp24
579	Galb1-4GlcNAcb1-3Galb1-4GlcNAcb1-3Galb1-4GlcNAcb1-3Galb1-4GlcNAcb1-2Mana1-6(Galb1-4GlcNAcb1-3Galb1-4GlcNAcb1-3Galb1-4GlcNAcb1-3Galb1-4GlcNAcb1-2Mana1-3)Manb1-4GlcNAcb1-4(Fuca1-6)GlcNAcb-Sp24
580	GlcNAcb1-3Galb1-4GlcNAcb1-3Galb1-4GlcNAcb1-3Galb1-4GlcNAcb1-3Galb1-4GlcNAcb1-2Mana1-6(GlcNAcb1-3Galb1-4GlcNAcb1-3Galb1-4GlcNAcb1-3Galb1-4GlcNAcb1-3Galb1-4GlcNAcb1-2Mana1-3)Manb1-4GlcNAcb1-4(Fuca1-6)GlcNAcb-Sp19
581	Galb1-4GlcNAcb1-3Galb1-4GlcNAcb1-3Galb1-4GlcNAcb1-3Galb1-4GlcNAcb1-3Galb1-4GlcNAcb1-2Mana1-6(Galb1-4GlcNAcb1-3Galb1-4GlcNAcb1-3Galb1-4GlcNAcb1-3Galb1-4GlcNAcb1-3Galb1-4GlcNAcb1-2Mana1-3)Manb1-4GlcNAcb1-4(Fuca1-6)GlcNAcb-Sp19
582	Galb1-4GlcNAcb1-3Galb1-4GlcNAcb1-6(Galb1-4GlcNAcb1-3Galb1-4GlcNAb1-2)Mana1-6(Galb1-4GlcNAcb1-3Galb1-4GlcNAcb1-2Mana1-3)Manb1-4GlcNAcb1-4(Fuca1-6)GlcNAcb-Sp24
583	GlcNAcb1-3Galb1-4GlcNAcb1-3Galb1-4GlcNAcb1-6(GlcNAcb1-3Galb1-4GlcNAcb1-3Galb1-4GlcNAb1-2)Mana1-6(GlcNAcb1-3Galb1-4GlcNAcb1-3Galb1-4GlcNAcb1-2Mana1-3)Manb1-4GlcNAcb1-4(Fuca1-6)GlcNAcb-Sp24
584	Galb1-4GlcNAcb1-3Galb1-4GlcNAcb1-3Galb1-4GlcNAcb1-6(Galb1-4GlcNAcb1-3Galb1-4GlcNAcb1-3Galb1-4GlcNAb1-2)Mana1-6(Galb1-4GlcNAcb1-3Galb1-4GlcNAcb1-3Galb1-4GlcNAcb1-2Mana1-3)Manb1-4GlcNAcb1-4(Fuca1-6)GlcNAcb-Sp24
585	GlcNAcb1-3Galb1-4GlcNAcb1-3Galb1-4GlcNAcb1-3Galb1-4GlcNAcb1-6(GlcNAcb1-3Galb1-4GlcNAcb1-3Galb1-4GlcNAcb1-3Galb1-4GlcNAb1-2)Mana1-6(GlcNAcb1-3Galb1-4GlcNAcb1-3Galb1-4GlcNAcb1-3Galb1-4GlcNAcb1-2Mana1-3)Manb1-4GlcNAcb1-4(Fuca1-6)GlcNAcb-Sp24
586	Galb1-4GlcNAcb1-3Galb1-4GlcNAcb1-3Galb1-4GlcNAcb1-3Galb1-4GlcNAcb1-6(Galb1-4GlcNAcb1-3Galb1-4GlcNAcb1-3Galb1-4GlcNAcb1-3Galb1-4GlcNAb1-2)Mana1-6(Galb1-4GlcNAcb1-3Galb1-4GlcNAcb1-3Galb1-4GlcNAcb1-3Galb1-4GlcNAcb1-2Mana1-3)Manb1-4GlcNAcb1-4(Fuca1-6)GlcNAcb-Sp24

Chart Number	Structure on Masterlist
587	GlcNAcb1-3Galb1-4GlcNAcb1-3Galb1-4GlcNAcb1-3Galb1-4GlcNAcb1-3Galb1-4GlcNAcb1-6(GlcNAcb1-3Galb1-4GlcNAcb1-3Galb1-4GlcNAcb1-3Galb1-4GlcNAcb1-3Galb1-4GlcNAb1-2)Mana1-6(GlcNAcb1-3Galb1-4GlcNAcb1-3Galb1-4GlcNAcb1-3Galb1-4GlcNAcb1-3Galb1-4GlcNAcb1-2Mana1-3)Manb1-4GlcNAcb1-4(Fuca1-6)GlcNAcb-Sp24
588	Galb1-4GlcNAcb1-3Galb1-4GlcNAcb1-3Galb1-4GlcNAcb1-3Galb1-4GlcNAcb1-3Galb1-4GlcNAcb1-6(Galb1-4GlcNAcb1-3Galb1-4GlcNAcb1-3Galb1-4GlcNAcb1-3Galb1-4GlcNAcb1-3Galb1-4GlcNAb1-2)Mana1-6(Galb1-4GlcNAcb1-3Galb1-4GlcNAcb1-3Galb1-4GlcNAcb1-3Galb1-4GlcNAcb1-3Galb1-4GlcNAcb1-2Mana1-3)Manb1-4GlcNAcb1-4(Fuca1-6)GlcNAcb-Sp24
589	Galb1-4GlcNAcb1-3Galb1-4GlcNAcb1-3GalNAca-Sp14
590	Galb1-4GlcNAcb1-3Galb1-4GlcNAcb1-6(Galb1-3)GalNAca-Sp14
591	Galb1-4GlcNAcb1-3Galb1-4GlcNAcb1-6(Galb1-4GlcNAcb1-3Galb1-4GlcNAcb1-3)GalNAca-Sp14
592	Neu5Aca2-3Galb1-4GlcNAcb1-3Galb1-4GlcNAcb1-3GalNAca-Sp14
593	GlcNAcb1-3Galb1-4GlcNAcb1-3GalNAca-Sp14
594	GlcNAcb1-3Galb1-4GlcNAcb1-6(Galb1-3)GalNAca-Sp14
595	GlcNAcb1-3Galb1-4GlcNAcb1-6(GlcNAcb1-3Galb1-4GlcNAcb1-3)GalNAca-Sp14
596	Neu5Aca2-3Galb1-4GlcNAcb1-3Galb1-4GlcNAcb1-6(Neu5Aca2-3Galb1-4GlcNAcb1-3Galb1-4GlcNAcb1-3)GalNAca-Sp14
597	Neu5Aca2-6Galb1-4GlcNAcb1-3Galb1-4GlcNAcb1-3GalNAca-Sp14
598	GlcNAcb1-3Galb1-4GlcNAcb1-3Galb1-4GlcNAcb1-3GalNAca-Sp14
599	Galb1-4GlcNAcb1-3Galb1-3GalNAca-Sp14
600	Neu5Aca2-3Galb1-4GlcNAcb1-3Galb1-4GlcNAcb1-6(Galb1-3)GalNAca-Sp14
601	Neu5Aca2-6Galb1-4GlcNAcb1-3Galb1-4GlcNAcb1-6(Galb1-3)GalNAca-Sp14
602	Neu5Aca2-6Galb1-4GlcNAcb1-6(Galb1-3)GalNAca-Sp14
603	Neu5Aca2-3Galb1-4GlcNAcb1-3Galb1-4GlcNAcb1-2Mana1-6(Neu5Aca2-3Galb1-4GlcNAcb1-3Galb1-4GlcNAcb1-2Mana1-3)Manb1-4GlcNAcb1-4GlcNAcb-Sp12
604	GlcNAcb1-6(Neu5Aca2-3Galb1-3)GalNAca-Sp14
605	Neu5Aca2-6Galb1-4GlcNAcb1-3Galb1-4GlcNAcb1-6(Neu5Aca2-6Galb1-4GlcNAcb1-3Galb1-4GlcNAcb1-3)GalNAca-Sp14
606	Neu5Aca2-6Galb1-4GlcNAcb1-3Galb1-4GlcNAcb1-3Galb1-4GlcNAcb1-2Mana1-6(Neu5Aca2-6Galb1-4GlcNAcb1-3Galb1-4GlcNAcb1-3Galb1-4GlcNAcb1-2Mana1-3)Manb1-4GlcNAcb1-4GlcNAcb-Sp12
607	Neu5Aca2-3Galb1-4GlcNAcb1-3Galb1-4GlcNAcb1-3Galb1-4GlcNAcb1-2Mana1-6(Neu5Aca2-3Galb1-4GlcNAcb1-3Galb1-4GlcNAcb1-3Galb1-4GlcNAcb1-2Mana1-3)Manb1-4GlcNAcb1-4GlcNAcb-Sp12
608	Neu5Aca2-6Galb1-4GlcNAcb1-3Galb1-4GlcNAcb1-2Mana1-6(Neu5Aca2-6Galb1-4GlcNAcb1-3Galb1-4GlcNAcb1-2Mana1-3)Manb1-4GlcNAcb1-4GlcNAcb-Sp12
609	GlcNAcb1-3Fuca-Sp21
610	Galb1-3GalNAcb1-4(Neu5Aca2-8Neu5Aca2-8Neu5Aca2-3)Galb1-4Glcb-Sp21

Sp0	$CH_2CH_2NH_2$
Sp8	$CH_2CH_2CH_2NH_2$
Sp9	$CH_2CH_2CH_2CH_2CH_2NH_2$
Sp10	$NHCOCH_2NH$
Sp11	$OCH_2C_6H_4$-p-$NHCOCH_2NH$
Sp12	Asparagine
Sp13	Glycine
Sp14	Threonine
Sp15	Serine
Sp16	PNP ($OC_6H_4NH_2$)
Sp17	$OCH_2C_6H_4NH_2$
Sp18	$O(CH_2)_3NHCO(CH_2)_5NH_2$
Sp19	EN or NK
Sp20	GENR
Sp21	-N(CH3)-O-(CH2)2-NH2
Sp22	NST
Sp23	(OCH2CH2)6NH2
Sp24	KVANKT
Sp25	VANK
MDPLys	Mur-L-Ala-D-iGlnb-(CH2)4NH2
