# Supplementary figures and images for: Host Tissue and Glycan Binding Specificities of Avian Viral Attachment Proteins Using Novel Avian Tissue Microarrays
Source: PLoS One. 2015 Jun 2;10(6):e0128893. doi: 10.1371/journal.pone.0128893 (PMC4452732; doi:10.1371/journal.pone.0128893)

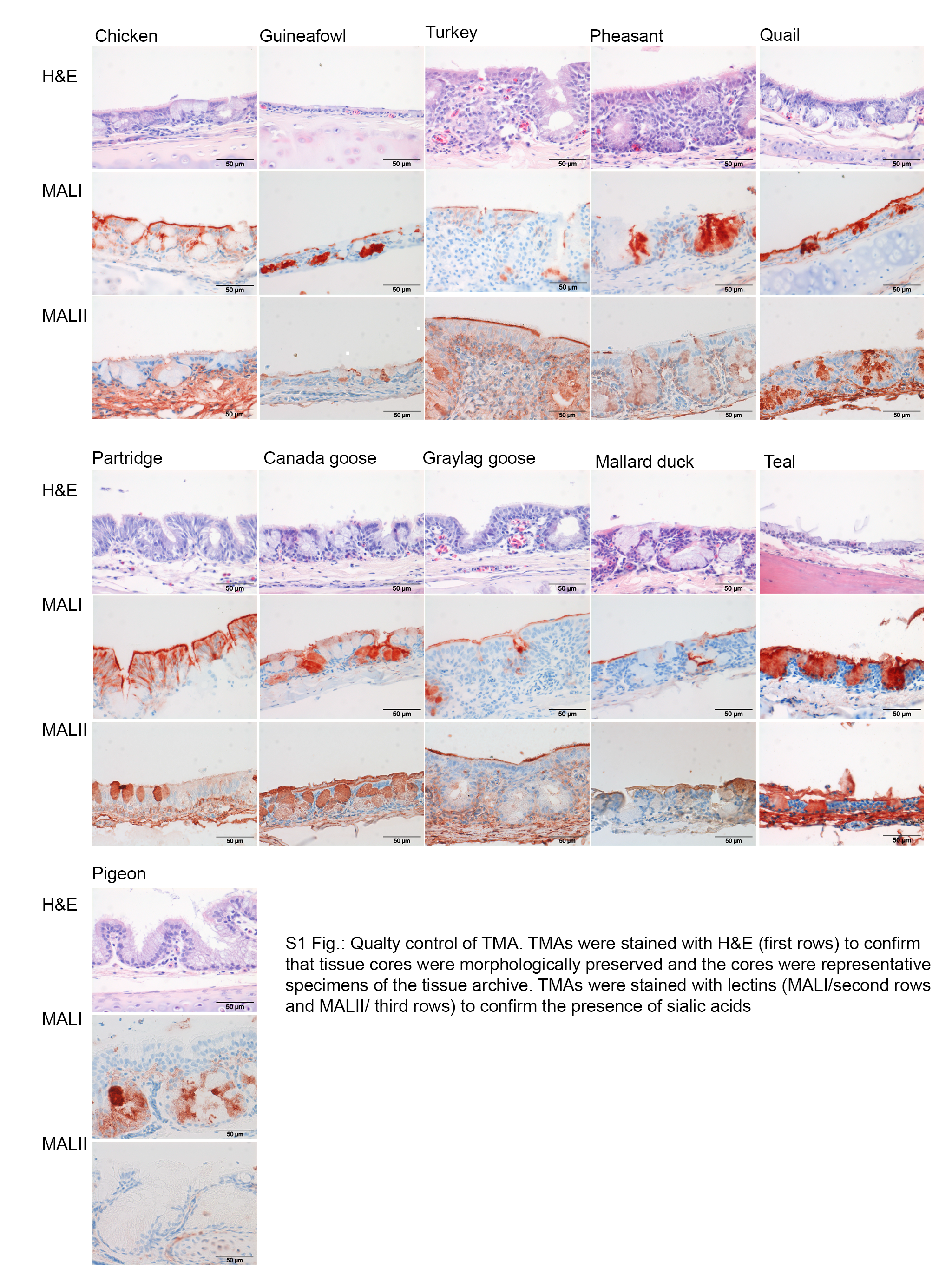

Supplement: S1 Fig — TMAs were stained with H&E (first rows) to confirm that tissue cores were morphologically preserved and the cores were representative specimens of the tissue archive. TMAs were stained with lectins (MALI/second rows and MALII/ third rows) to confirm the presence of sialic acids. (TIF) [file pone.0128893.s002.tif]

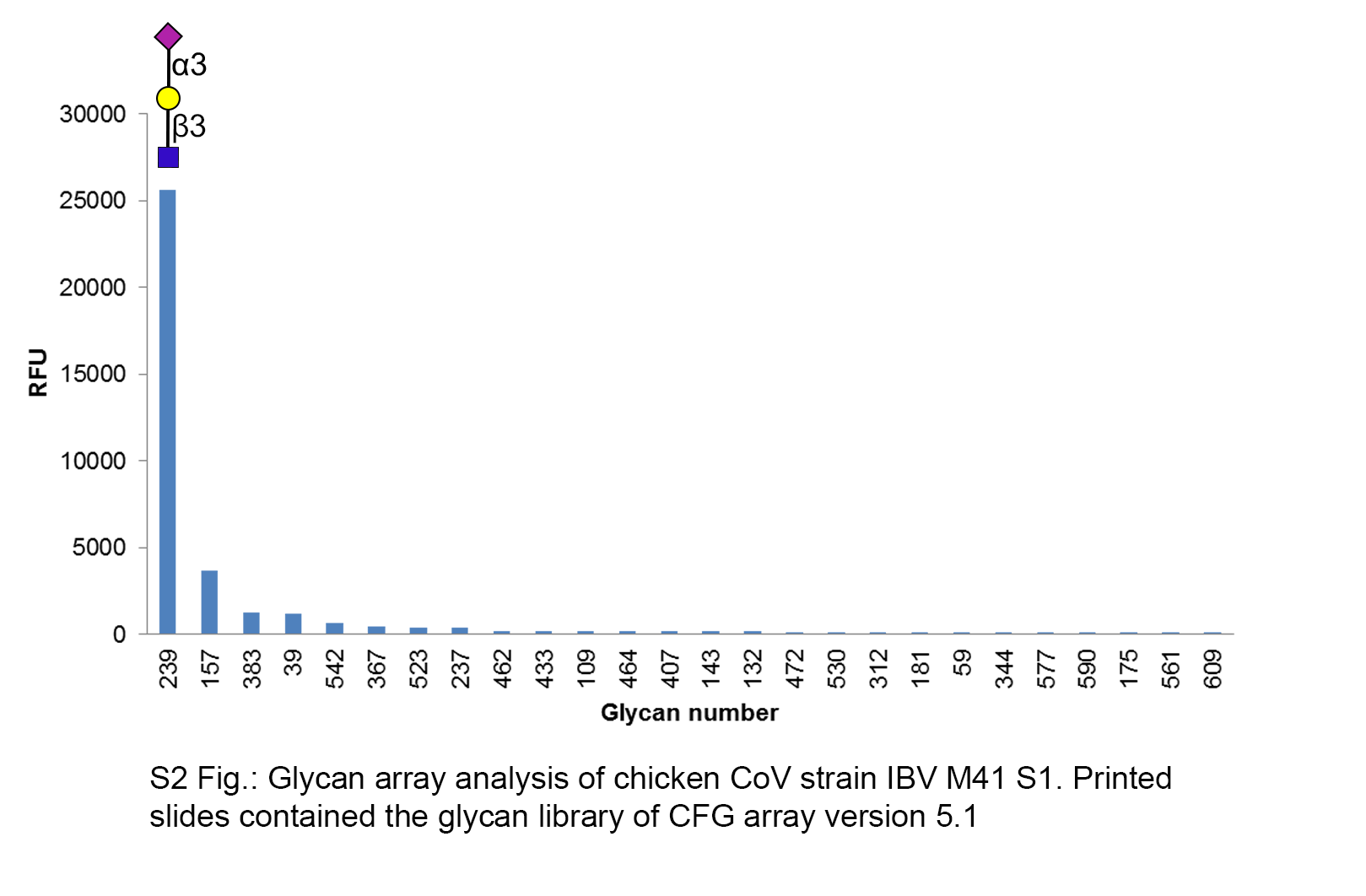

Supplement: S2 Fig — Printed slides contained the glycan library of CFG array version 5.1 (TIF) [file pone.0128893.s003.tif]
